# Plasticization of a Semicrystalline Metallosupramolecular Polymer Network

**DOI:** 10.1021/acspolymersau.2c00044

**Published:** 2022-11-10

**Authors:** Franziska Marx, Subhajit Pal, Julien Sautaux, Nazim Pallab, Grégory Stoclet, Christoph Weder, Stephen Schrettl

**Affiliations:** † Adolphe Merkle Institute, University of Fribourg, Chemin des Verdiers 4, Fribourg 1700, Switzerland; ‡ CNRS, INRAE, Centrale Lille, UMR 8207 - UMET - Unité Matériaux et Transformations, Univ. Lille, Lille F-59000, France; § TUM School of Life Sciences, Technical University of Munich, Maximus-von-Imhof-Forum 2, Freising 85354, Germany

**Keywords:** supramolecular polymers, metal−ligand complexes, plasticization, glass transition temperature, mechanical properties, stimuli-responsive polymers, debonding on demand

## Abstract

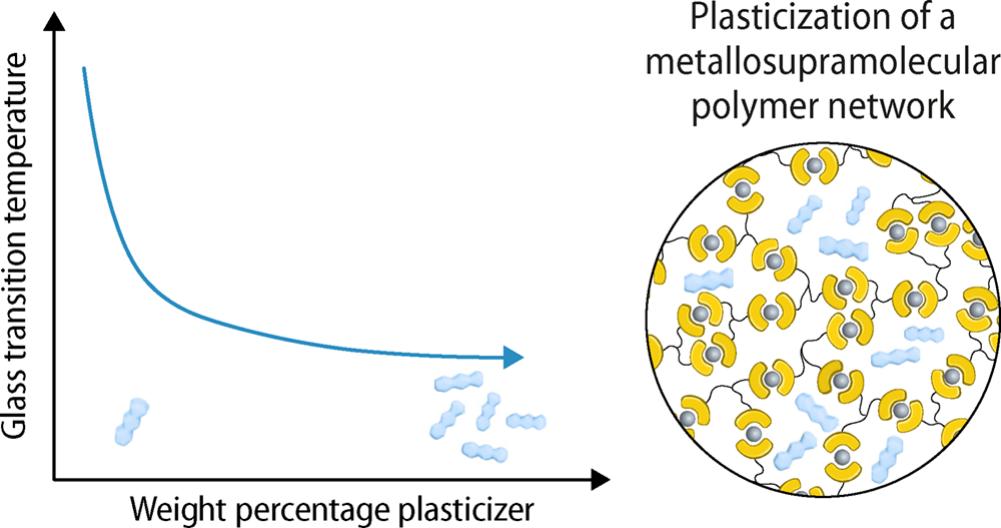

The assembly of ligand-functionalized (macro)monomers with suitable metal ions affords metallosupramolecular polymers (MSPs). On account of the reversible and dynamic nature of the metal–ligand complexes, these materials can be temporarily (dis-)assembled upon exposure to a suitable stimulus, and this effect can be exploited to heal damaged samples, to facilitate processing and recycling, or to enable reversible adhesion. We here report on the plasticization of a semicrystalline, stimuli-responsive MSP network that was assembled by combining a low-molecular-weight building block carrying three 2,6-bis(1′-methylbenzimidazolyl) pyridine (Mebip) ligands and zinc bis(trifluoromethylsulfonyl)imide (Zn(NTf_2_)_2_). The pristine material exhibits high melting (*T*_m_ = 230 °C) and glass transition (*T*_g_ ≈ 157 °C) temperatures and offers robust mechanical properties between these temperatures. We show that this regime can be substantially extended through plasticization. To achieve this, the MSP network was blended with diisodecyl phthalate. The weight fraction of this plasticizer was systematically varied, and the thermal and mechanical properties of the resulting materials were investigated. We show that the *T*_g_ can be lowered by more than 60 °C and the toughness above the *T*_g_ is considerably increased.

## Introduction

Supramolecular, polymer-like chains or networks are formed when (macro)monomeric building blocks are assembled by means of directional, noncovalent interactions.^1−3^ Binding motifs that can form hydrogen-bonding interactions, host–guest interactions, or those that allow for the formation of metal–ligand complexes have been successfully employed to assemble such materials.^4−6^ A widely employed design approach involves the assembly of telechelic building blocks featuring two self-complementary binding motifs at the termini. While this framework affords linear chain-extended polymers, it is also possible to create supramolecular polymer networks from multifunctional monomers that feature more than two binding motifs.^7−9^ In contrast to classic polymers or polymer networks, in which the individual monomers are covalently linked, the supramolecular counterparts can be readily disassembled into the constituting building blocks by applying a suitable external stimulus such as light or heat that sufficiently weakens the noncovalent interactions.^10−12^ The reversibility of the assembly renders supramolecular polymers an attractive material class and imparts them with ease of processing and recyclability, as well as intriguing stimuli-responsive behaviors. Particularly drastic responses to external stimuli are observed for materials that are assembled from building blocks having a low molecular weight. The disassembly can be, for example, accompanied by a substantial viscosity decrease, which translates into a distinct advantage when considering aspects of processing or recycling.^13,14^ Moreover, the dynamic response of supramolecular polymers to external stimuli can render them healable,^15−21^ enables reversible adhesion,^22−25^ and imparts shape-memory effects^26−29^ and many other types of functional responses.^10−12^

The bulk mechanical properties and stimuli-responsive behavior of supramolecular polymers are significantly influenced by the nature of the molecular or macromolecular core that links the binding motifs and by phase separation effects, i.e., the formation of crystalline or glassy domains of the binding motifs, which act as additional physical cross-links.^30−35^ There are many examples of supramolecular building blocks that contain telechelic linkers that are amorphous with a glass transition temperature (*T*_g_) significantly below room temperature.^33−37^ The supramolecular self-assembly of such building blocks into polymers is then primarily governed by the association between the binding motifs, but such materials typically display a low stiffness and strength, similar to (thermoplastic) elastomers. When building blocks are employed that form a glassy phase with a high *T*_g_ or semicrystalline domains with a high melting temperature (*T*_m_), the mechanical properties are often governed by the characteristics of the polymer, which can in turn hamper the association of binding motifs and the chain extension.^38−40^

**Figure 1:**
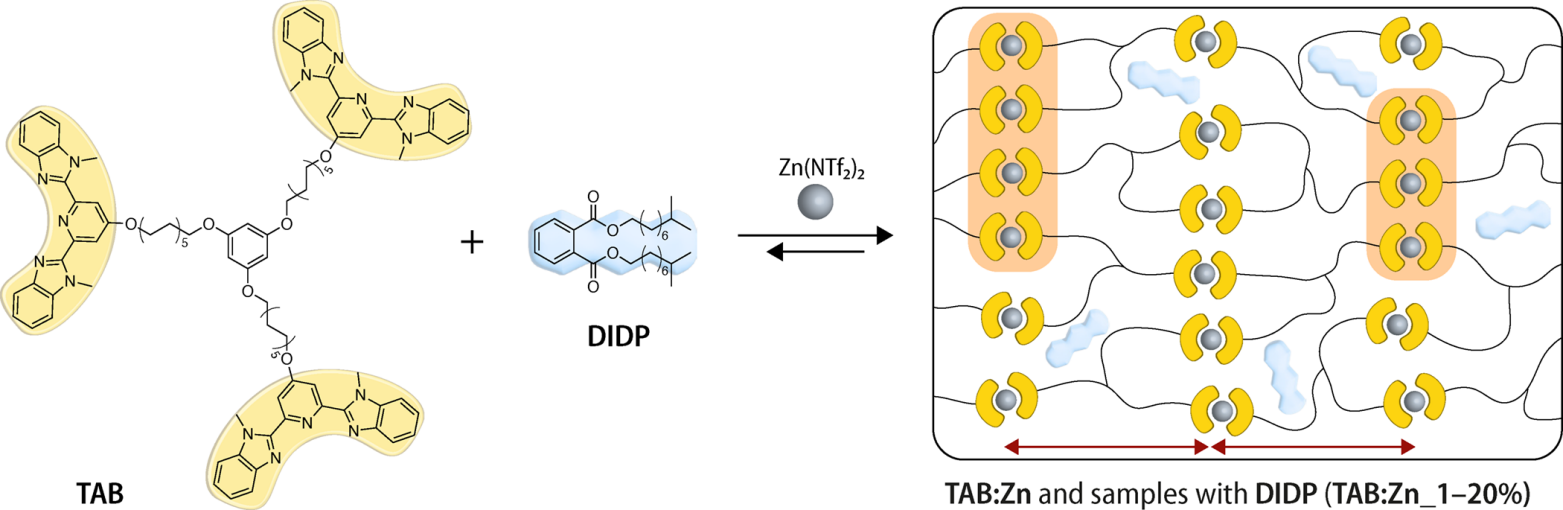
Chemical structure of the trifunctional building block **TAB** and the diisodecyl phthalate (**DIDP**) plasticizer and schematic representation of the assembly into a metallosupramolecular polymer (MSP) network (**TAB:Zn**) with a stoichiometric amount of zinc(*II*)bis(trifluoromethanesulfonyl)imide (Zn(NTf_2_)_2_). Different weight fractions of **DIDP** were used to produce the plasticized **TAB:Zn_1–20%** samples. The MSP network features a phase-separated lamellar structure (red arrows) with crystalline domains (orange) of the metal–ligand complexes, and the plasticizer resides in the amorphous regions of the material.

Supramolecular polymers with a high stiffness at room temperature can also be accessed from low-molecular-weight building blocks that feature a high density of binding motifs, e.g., hydrogen-bonding 2-ureido-4[1H]-pyrimidinones^41^ or isophthalic acid–pyridine interactions.^14^ The assembly of such multifunctional building blocks furnished amorphous polymeric materials that display a very high stiffness, viscoelastic flow in the melt, and complete disassembly in solution, but they are generally very brittle.^14,41^ We recently reported a trifunctional low-molecular-weight building block (**TAB**) comprised of a trialkylbenzene core equipped with the well-established 2,6-bis(1′-methylbenzimidazolyl)pyridine (Mebip) ligand (Figure 1).^42^ The latter is known to form metal–ligand complexes with different transition metal or lanthanoid ions,^43−46^ and the combination of **TAB** with stoichiometric amounts of zinc triflimide (Zn(NTf_2_)_2_) furnishes a stiff, semicrystalline metallosupramolecular polymer (MSP) network (**TAB:Zn**) with high melting (*T*_m_ > 200 °C) and glass transition temperatures (*T*_g_ > 140 °C). The mechanical properties of this stiff and rather brittle MSP depend on the processing conditions, which greatly impact the crystallinity. The properties can also be varied over a large range via the co-assembly with a building block featuring the same ligand but a low-*T*_g_ telechelic core. On account of microphase separation into hard and soft domains, the resulting materials display, depending on the composition, higher strength, toughness, or failure strain than either of the individual MSPs.

Here, we report that the thermal and mechanical properties of the MSP network **TAB:Zn** can also be modified by conventional plasticization. This strategy is commonly employed for conventional polymers,^47−50^ but it has been rarely applied to supramolecular polymers.^51^ We surmised that the MSP formed from **TAB** and Zn(NTf_2_)_2_ is an ideal testbed to explore the plasticization of a supramolecular polymer, since low-molecular-weight additives can be expected to modify interchain interactions in the amorphous regions of this MSP and effectively lower the *T*_g_ of this phase without affecting the crystalline domains.^52,53^ Leibler and co-workers previously reported a hydrogen-bonded supramolecular polymer that was plasticized with ca. 11 wt % of dodecane.^51^ The plasticizer lowered the *T*_g_ from 28 to 8 °C and increased the extensibility of the material from 350% at 90 °C to above 500% at room temperature. We show here that the addition of diisodecyl phthalate (**DIDP**) can lower the *T*_g_ of **TAB:Zn** by more than 60 °C. Intriguingly, a high stiffness, strength, and extensibility are observed above *T*_g_ for the plasticized materials, which corroborates that the addition of additives is a viable pathway for tuning the bulk properties of supramolecular polymers. The heat-induced disassembly of the MSP network moreover enables its usage as a reversible debonding-on-demand adhesive with a high stiffness and strength.

## Results and Discussion

The trifunctional low-molecular-weight building block equipped with Mebip ligands (**TAB**, Figure 1) was prepared from commercially available 1,3,5-trihydroxybenzene in three steps (Figure S1). We adapted a previously reported procedure^42^ and carried out the Williamson ether synthesis to access 1,3,5-tris(undec-10-en-1-yloxy)benzene from 1,3,5-trihydroxybenzene and 11-bromo-1-undecene in the presence of 18-crown-6. 1,3,5-Tris(undec-10-en-1-yloxy)benzene was subsequently converted into the corresponding alcohol derivative by hydroboration and oxidation. The reaction with 2,6-bis(1′-methylbenzimidazolyl) pyridin-4-ol under Mitsunobu conditions finally afforded **TAB** as an analytically pure, semicrystalline powder in an overall yield of 36% (see the Supporting Information for details). After screening different plasticizers (see the Experimental Section for details), commercially available **DIDP** was chosen for in-depth studies, due to its apparent miscibility with the MSP and its relatively high boiling temperature (370 °C), which is beneficial for the processing of the MSP at elevated temperatures and the retention of the plasticizer in the material.

**Figure 2:**
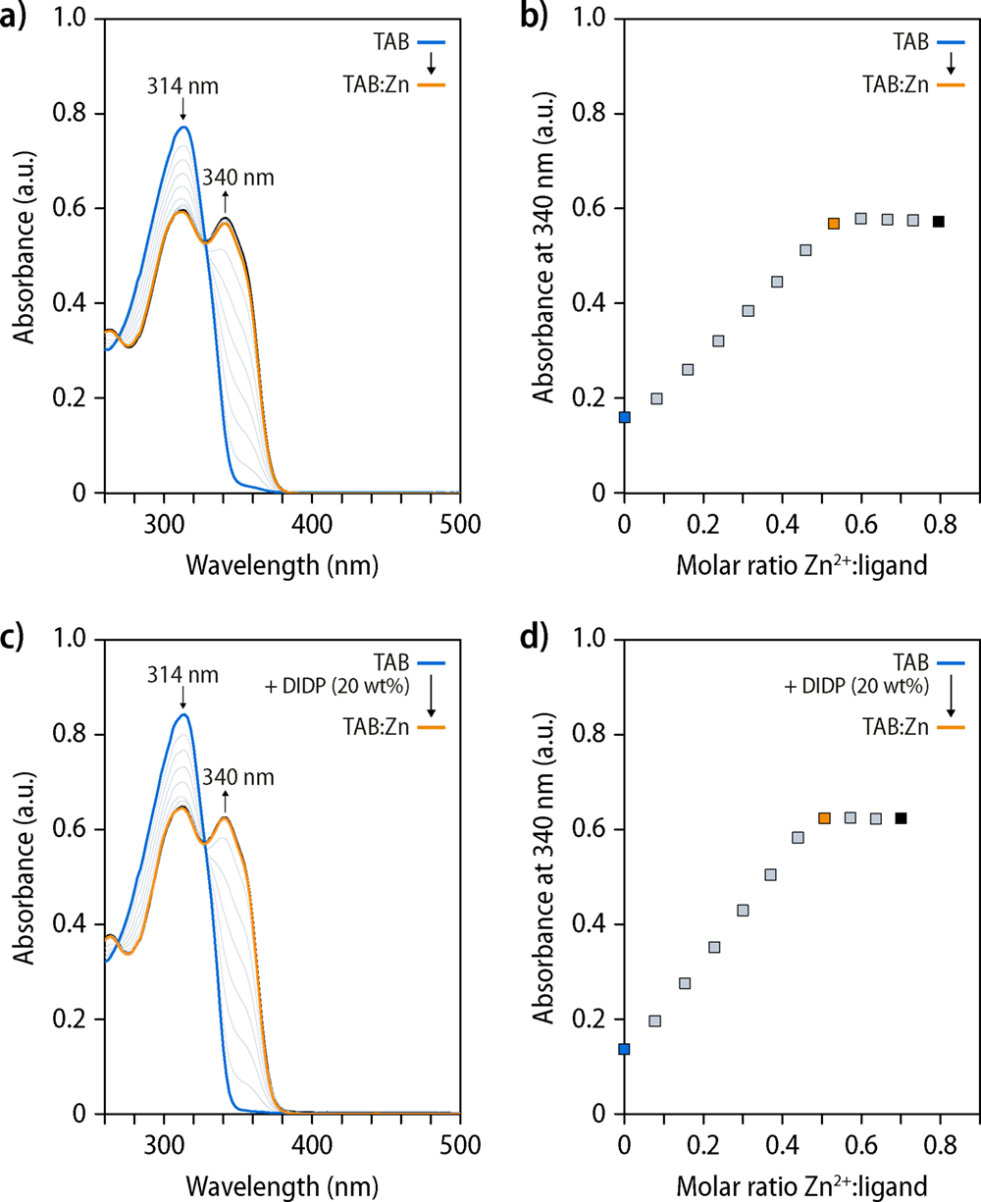
Spectrophotometric titrations of **TAB** with Zn(NTf_2_)_2_. (a) UV–vis absorption spectra recorded during the titration of a solution of **TAB** with Zn(NTf_2_)_2_ and (b) plot of the absorbance at 340 nm as a function of the M/L ratio. Data were acquired upon adding 25 μL aliquots of a solution of Zn(NTf_2_)_2_ and **TAB** in a 9:1 *v*/*v* CHCl_3_/CH_3_CN mixture (132 and 6.71 μM) to a solution of **TAB** (6.71 μM) in the same solvent mixture. (c) UV–vis absorption spectra recorded during the titration of a solution of **TAB** containing 20 wt % of diisodecyl phthalate (**DIDP**) with respect to **TAB** with Zn(NTf_2_)_2_ and (d) plot of the absorbance at 340 nm as a function of the M/L ratio. Data were acquired upon adding 25 μL aliquots of a solution of Zn(NTf_2_)_2_ (146 μM) and **TAB** (7.72 μM) in a 9:1 *v*/*v* CHCl_3_/CH_3_CN mixture to a solution of **TAB** (7.72 μM) and **DIDP** in the same solvent mixture.

To probe the formation of the metal–ligand complexes in the presence of the plasticizer, spectrophotometric titrations were carried out with **TAB** solutions (*c* = ca. 7 μmol L^–1^) in a chloroform/acetonitrile mixture (9:1 *v*/*v*) in the absence (Figure 2a,b) and presence of 20 wt % of **DIDP** relative to **TAB** (Figure 2c,d). Thus, aliquots of a solution of zinc(II) bis(trifluoromethanesulfonyl)imide (Zn(NTf_2_)_2_) were added to the **TAB** solutions and the changes in the UV–vis absorption spectra were monitored at wavelengths correlated with the diagnostic bands of the metal–ligand complex.^42,46^ The spectra of solutions with and without **DIDP** show that the absorption band at 314 nm associated with the Mebip ligand is shifted to 340 nm upon addition of Zn(NTf_2_)_2_, indicating the formation of the metal–ligand complexes. The corresponding plots of the absorption at 340 nm against the Zn^2+^/ligand ratio confirm the formation of 1:2 coordination complexes at a stoichiometric metal-to-ligand ratio, even in the presence of **DIDP** (Figure 2b,d). Moreover, UV–vis absorption spectra recorded upon addition of **DIDP** to solutions containing a stoichiometric mixture (3:2) of Zn(NTf_2_)_2_ and **TAB** show only a dilution effect (Figure S2a), and no complex formation was observed upon addition of aliquots of a solution of Zn(NTf_2_)_2_ to a solution of **DIDP** (Figure S2b). Thus, the data confirm unequivocally that the plasticizer does not interfere with the metal–ligand complex formation.

To explore the effect of the plasticizer on the thermal and mechanical properties of the MSP network, samples of **TAB:Zn** with a 1:2 metal-to-ligand (M/L) stoichiometry without and with the plasticizer were prepared by solvent casting from solution. Solutions of **TAB** (*c* = 0.012 mmol mL^–1^) in chloroform were mixed with acetonitrile solutions containing a stoichiometric amount of Zn(NTf_2_)_2_ (*c* = 0.045 mmol mL^–1^), and the mixtures were cast into poly(tetra fluoroethylene) molds. For plasticized samples (**TAB:Zn_1–20%**), chloroform solutions (2 mL) containing either 1, 2.5, 5, 10, or 20 wt % of **DIDP** with respect to the MSP were added to stoichiometric mixtures of **TAB** and the zinc salt. Such mixtures were stirred for 30 min prior to solvent casting. After solvent evaporation, samples were dried at 50 °C for 24 h in vacuo to furnish rigid, transparent films with appreciable mechanical properties. To explore if samples can be thermally processed at elevated temperatures without loss of the plasticizer, the thermal properties of **TAB:Zn** and **TAB:Zn_1–20%** were investigated by thermogravimetric analysis (TGA). The TGA trace of **TAB:Zn** indicates a 5% (*w*/*w*) mass loss above 350 °C, while an onset of mass loss is observed at ca. 200 °C for samples of **TAB:Zn_1–20%** (Figure S3a). A comparison with the TGA trace of neat **DIDP** suggests that the lower onset temperature at which mass loss was observed for the **TAB:Zn_1–20%** samples corresponds to the evaporation of **DIDP**. To elucidate the extent of mass lost at 220 °C, i.e., the temperature at which the cast films were further processed (vide infra), samples of neat **DIDP**, **TAB:Zn**, **TAB:Zn_1%**, and **TAB:Zn_10%** were subjected to isothermal measurements at this temperature (Figure S3c). The recorded traces indicate that a slow weight reduction occurs for plasticized samples at this temperature, but the mass loss is limited to below 0.5 wt % over a course of 2 min, suggesting that processing is possible without a significant reduction of the plasticizer content.

To process the (plasticized) MSPs into films with a uniform thickness (ca. 170–200 μm), solvent-cast films were compression-molded in a hot press at 220 °C (see the Experimental Section for a detailed description). Samples of **TAB:Zn** and **TAB:Zn_1–5%** were processed at this temperature with a pressure of 6 t for 45 s, while a pressure of 4 t and a compression for 30 s were sufficient to obtain uniform **TAB:Zn_10–20%** films. Unless noted otherwise, samples were slowly cooled between the steel plates of the hot press over a course of 45 min. While the compression-molded films of **TAB:Zn** and **TAB:Zn_1–5%** were transparent, films with 10 wt % or more of **DIDP** appeared turbid and their surface was greasy (Figure S3), indicating that processing at this temperature leads to plasticizer sweating. Indeed, a comparison of the TGA traces of solvent-cast and compression-molded samples corroborates the loss of plasticizer for **TAB:Zn_10–20%** samples (Figure S4b,d), whereas the traces of **TAB:Zn_1–5%** recorded before and after compression molding are comparable. Thus, the maximum concentration of **DIDP** that can be incorporated into **TAB:Zn** appears to be limited to <10 wt %, and attempts to incorporate larger amounts lead to macrophase separation. While **TAB:Zn_10–20%** films were nonetheless characterized, the absolute weight fraction of **DIDP** in these samples is lower than the nominal concentration quoted.

**Figure 3:**
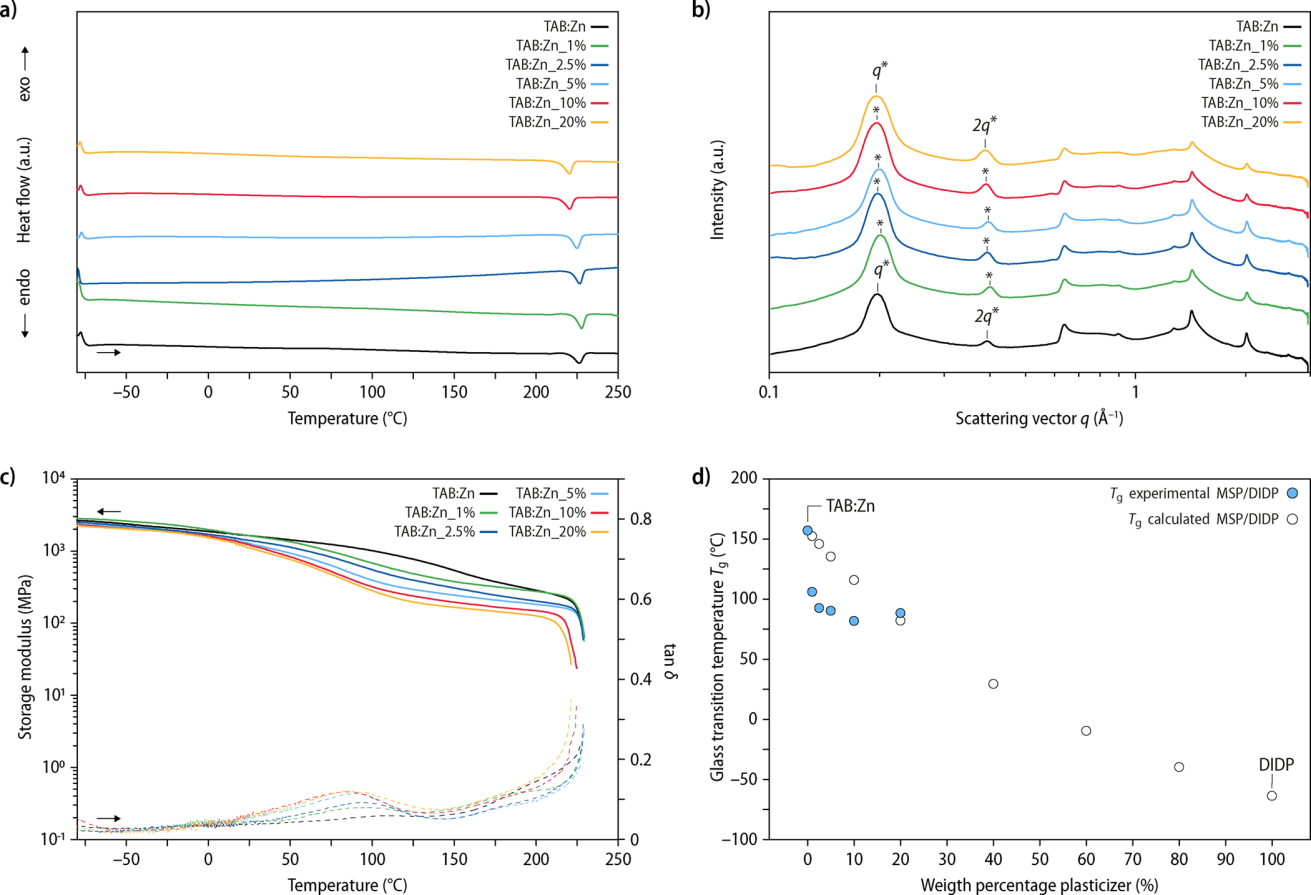
Thermal, structural, and mechanical characterization of compression-molded and slowly cooled samples of **TAB:Zn** and **TAB:Zn_1–20%**. Shown are comparisons of representative (a) differential scanning calorimetry (DSC) traces (first heating scans), (b) small- and wide-angle X-ray scattering (SAXS/WAXS) profiles, and (c) dynamic mechanical analysis (DMA) traces (solid lines: storage modulus, dashed lines: tan δ). (d) Plot of the experimentally determined and calculated glass transition temperature (*T*_g_) against the weight fraction of the added plasticizer in the metallosupramolecular polymer. A previously reported *T*_g_ value of DIDP that was determined by DMA measurements^54^ was used for the calculation of expected *T*_g_ values with the Fox equation. The shown DSC traces and SAXS/WAXS scattering profiles are vertically shifted for clarity.

Compression-molded and solvent-cast samples of **TAB:Zn** and **TAB:Zn_1–20%** were characterized by differential scanning calorimetry (DSC). The first and second DSC heating traces of compression-molded **TAB:Zn** samples display a reversible melting transition (*T*_m_) at ca. 231 °C, and a corresponding crystallization transition (*T*_c_) is observed in the cooling trace at ca. 180 °C (Figures 3a and S5 and Tables 1 and S1); however, no glass transition is discernible. The DSC traces of **TAB:Zn_1–5%** mirror those of the neat **TAB:Zn**, whereas the traces of samples containing 10 wt % or more **DIDP** display slightly reduced *T*_m_ (ca. 220 °C) and *T*_c_ (ca. 176 °C) values (Figures 3a and S5 and Tables 1 and S1). A comparison of the melting enthalpies shows that this value does not change significantly; i.e., the extent of crystallinity in all samples remains the same throughout repeated heating and cooling experiments (Figure S4; Table S1). Moreover, a comparison of the first DSC heating traces of compression-molded and solvent-cast samples shows a minor increase in the *T*_m_ for the former, which is most pronounced for **TAB:Zn_5%** samples (Figures S5 and S6; Table S1). This observation is tentatively interpreted as an annealing effect, as previously observed for neat **TAB:Zn**,^42^ and corroborated by the fact that the first cooling and second heating traces of melt-processed samples mirror those of solvent-cast samples (Figures S5 and S6). The DSC measurements, hence, indicate that the semicrystalline nature of the MSP network is retained in the presence of the plasticizer, even though the melting and crystallization temperatures are slightly lower if the **DIDP** content is above 5 wt %. Moreover, taking the results from DSC and TGA measurements into account, the plasticizer appears to be largely retained in the MSP when samples are briefly heated above the onset of **DIDP** evaporation of ca. 200 °C, but prolonged heating at these temperatures leads to a gradual loss of the plasticizer.

**Table 1:** Overview of the Thermal and Mechanical Properties of **TAB:Zn** and **TAB:Zn_1–20%** Samples Plasticized with Different Amounts of Diisodecyl Phthalate

	*T*_m_	*T*_g_	failure temp.	storage modulus	Young’s modulus	tensile strength	strain at break	toughness
sample	(°C)^a^	(°C)^b^	(°C)^b^	*E*’ (25 °C; GPa)^b^	*E* (GPa)^c^	σ_u_ (MPa)^c^	ε_b_ (%)^c^	*U*_T_ (kJ m^–3^)^c^
TAB:Zn	231	157 ± 1^d^	230 ± 2	1.73 ± 0.16	1.29 ± 0.07	9 ± 1	0.8 ± 0.1	36 ± 11
TAB:Zn_1%	233	106 ± 3	232 ± 3	1.58 ± 0.04	1.35 ± 0.05	9 ± 1	0.8 ± 0.1	42 ± 11
TAB:Zn_2.5%	227	93 ± 3	230 ± 1	1.41 ± 0.17	1.16 ± 0.10	9 ± 2	1.0 ± 0.2	45 ± 17
TAB:Zn_5%	230	90 ± 2	230 ± 0	1.32 ± 0.21	0.95 ± 0.13	9 ± 1	0.9 ± 0.1	46 ± 15
TAB:Zn_10%	220	82 ± 2	229 ± 2	1.32 ± 0.28	0.93 ± 0.07	8.7 ± 0.4	1.0 ± 0.1	49 ± 34
TAB:Zn_20%	222	88 ± 3	224 ± 3	1.22 ± 0.23	n.d.	n.d.	n.d.	n.d.

a Measured by DSC with a heating rate of 10 °C min^–1^.

b Measured by DMA with a heating rate of 3 °C min^–1^. The *T*_g_ was determined from the local maximum in the tan δ traces. Values represent averages of *n* = 2–6 individual measurements ± standard deviation.

c Determined by stress–strain measurements at 25 °C with a strain rate of 1% min^–1^. Values represent averages of *n* = 3–6 individual measurements ± standard deviation.

d Measured on samples that were quenched by rapid cooling from a temperature of 230 °C. n.d., not determined.

In order to further elucidate the influence of **DIDP** on the morphology of the MSPs, melt-processed samples of **TAB:Zn** and **TAB:Zn_1–20%** were subjected to small- and wide-angle X-ray scattering (SAXS/WAXS) measurements (Figure 3b). The scattering profiles show that the microstructure of **TAB:Zn** remains essentially unchanged upon plasticization with **DIDP**. The scattering profiles of samples of **TAB:Zn** exhibit Bragg peaks at relative positions of q* and 2q*, indicative of a microphase-separated lamellar morphology that features a characteristic spacing of ca. 3.19 nm (Figure 3b; Table S2). In agreement with the DSC measurements, Bragg reflections at larger scattering vectors (*q* ≈ 0.6–2.3 Å^–1^) corroborate the presence of some degree of crystalline order. A comparison of the scattering profiles of **TAB:Zn_1–20%** samples indicates that neither the signals observed in the SAXS regime nor the peaks in the WAXS regime experience considerable changes upon plasticization (Figure 3b; Table S2). Additional SAXS measurements at smaller angles were carried out in order to explore if pure **DIDP** domains phase separate in compositions with high plasticizer concentrations, but the scattering profiles show no indication of a structure formation on larger length scales (Figure S7). These findings corroborate that the plasticizer primarily if not exclusively resides in the amorphous domains, without impacting the lamellar structuration or crystalline domains of the MSP.

The thermomechanical properties of **TAB:Zn** and **TAB:Zn_1–20%** samples were characterized by subjecting compression-molded films to dynamic mechanic analysis (DMA) measurements. The DMA trace of **TAB:Zn** displays a wide regime of high rigidity, with a storage modulus *E*’ of 2.53 ± 0.21 GPa at −80 °C and an *E*’ of ca. 1.73 ± 0.16 GPa at room temperature (Figure 3c; Table 1). The sample fails at 230 °C, i.e., at a temperature that coincides with the *T*_m_ established by DSC. The *E*’ vs temperature plots also show a slope change, which is particularly pronounced in samples that were rapidly cooled after processing (see the Supporting Information for details); a corresponding maximum in the tan δ curve at around 157 °C marks the *T*_g_ of the semicrystalline MSP network (Figure S8). The DMA traces of **TAB:Zn** samples that were cooled slowly after processing show no clear glass transition, and *E*’ remains high (above 200 MPa) until the samples melt, reflecting a higher degree of crystallinity. The DMA measurements of plasticized samples, which were all cooled slowly after processing, show that the storage modulus at −80 °C remains unchanged, irrespective of the **DIDP** content. However, the temperature at which the storage modulus of the material starts to drop is reduced, and the room-temperature modulus gradually decreases (Figure 3c; Table 1). The temperature at which the changes occur appears to level off above a **DIDP** content of 5 wt %, suggesting that the miscibility limit is reached at this plasticizer content. Moreover, the failure temperatures of **TAB:Zn** and **TAB:Zn_1–20%** samples are in excellent agreement with the melting transitions determined in DSC measurements (Figure 3c; Table 1), corroborating that the semicrystalline domains act as physical crosslinks that provide mechanical stability above the *T*_g_.

Importantly, the tan δ traces of plasticized samples show a distinct reduction of the *T*_g_ with an increasing weight fraction of **DIDP** (Figure 3c, dashed lines). While the neat MSP network features a *T*_g_ of ca. 157 °C (Figure S8), the latter is reduced to ca. 106 ± 3 °C for **TAB:Zn_1%** and 90 ± 2 °C for **TAB:Zn_5%** (Figures 3d and S9 and Table 1). Increasing the **DIDP** content beyond 5 wt % does not lead to a further reduction in *T*_g_, which suggests that the miscibility limit has been reached, and no further plasticization is possible.^55−58^ The extent of plasticization can be estimated by several relationships, including the Fox equation,^55^ which approximates samples’ *T*_g_ by considering the *T*_g_ values and weight fractions of the polymer matrix and the plasticizer (see the Supporting Information for details). Experimental results for **TAB:Zn_1–20%** were, hence, compared to the expected plasticization by using the *T*_g_ of **TAB:Zn** (ca. 157 °C) and a previously reported value for **DIDP** (*T*_g_ ≈ *–*64 °C).^54^ Intriguingly, the corresponding plot of the calculated *T*_g_ values against the weight fraction of the added plasticizer shows that the experimental values are significantly lower than expected for a **DIDP** content of up to 5 wt % (Figure 3d, circles), which is in stark contrast to reported *T*_g_ values for samples of poly(vinyl chloride) plasticized with **DIDP**(59) or di(2-ethylhexyl phthalate)^60^ (**DEHP**; *T*_g_ ≈ *–*85 °C)^54^ and the values calculated with the Fox equation (Figure S10). Even at the lowest plasticizer concentrations, for which one can assume complete miscibility, we observe a pronounced deviation from the behavior predicted based on the Fox equation. We speculate that this is due to the semicrystalline nature of the MSP network, which leads to an increase of the effective concentration of the plasticizer in the amorphous phase. Other pertinent factors may include larger differences in the heat capacities of the plasticizer and polymer, a high plasticizer efficiency, the impact of the plasticizer on the dynamics of metal–ligand complexes in the solid state, and the fundamentally different characteristics of supramolecular polymers.

**Figure 4:**
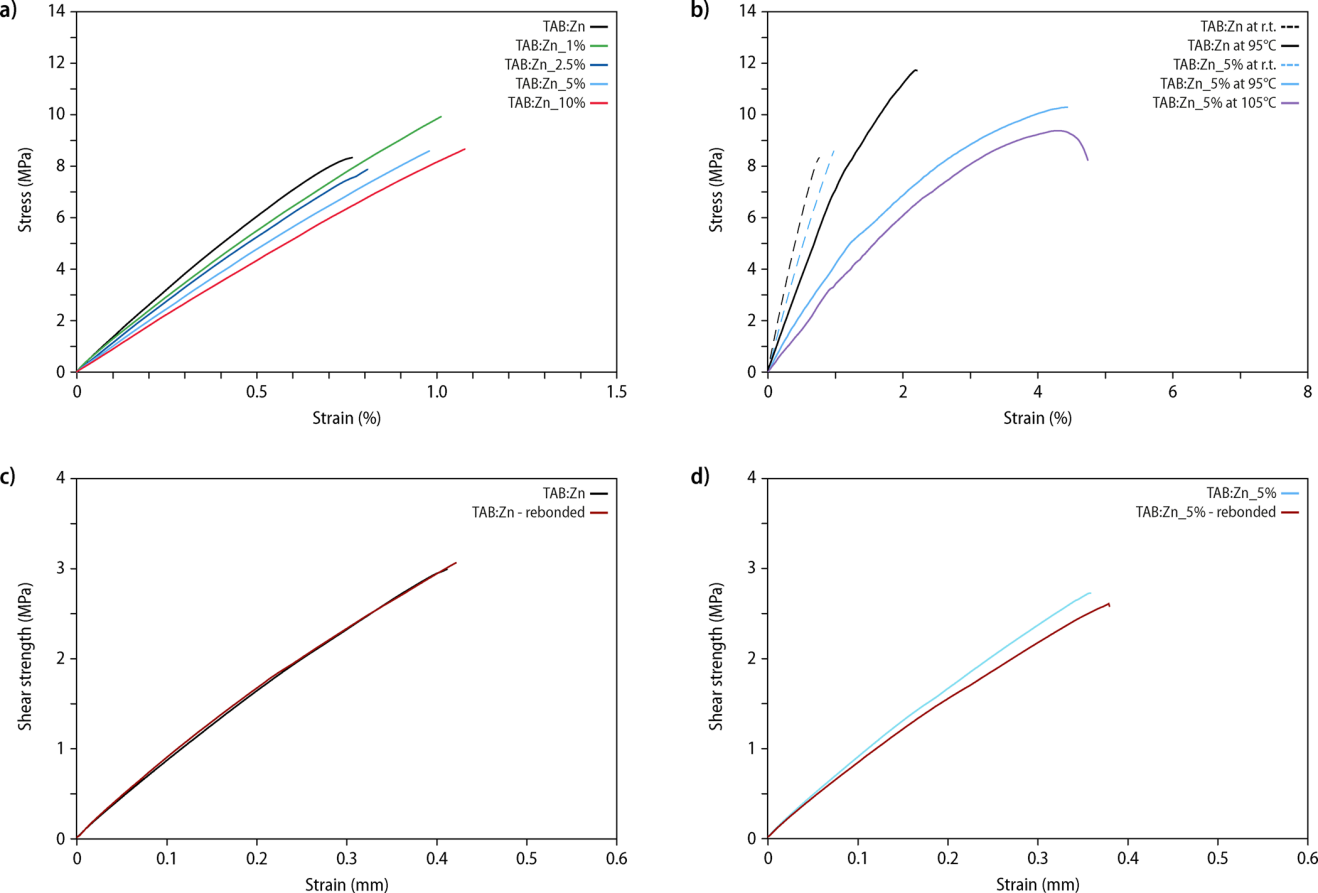
Mechanical characterization of compression-molded films of **TAB:Zn** and **TAB:Zn_1–20%** and shear tests of MSP-bonded single lap joints. (a) Comparison of representative stress–strain curves acquired by uniaxial tensile testing at 25 °C. (b) Comparison of the stress–strain curves of **TAB:Zn** and **TAB:Zn_5%** acquired at 25 °C (dashed lines) as well as 95 and 105 °C (solid lines). (c, d) Shear tests of stainless-steel single lap joint properties bonded with (c) **TAB:Zn** or (d) **TAB:Zn_5%**. Shown are representative shear strength vs strain curves for lap joints that were freshly bonded for 20 s at 230 °C and re-bonded under the same conditions after failure (red lines).

The mechanical properties of **TAB:Zn** and **TAB:Zn_1–20%** were further characterized by subjecting compression-molded films to uniaxial tensile tests. Tensile tests were first carried out at ambient temperature (Figures 4a and S11 and Table 1). The stress–strain curves of **TAB:Zn** films reflect the stiff and brittle nature of this material with a Young’s modulus (*E*) of ca. 1.29 ± 0.07 GPa, a tensile strength of 9 ± 1 MPa, and a strain at break of 0.8 ± 0.1% (Figure 4a; Table 1). In agreement with the DMA traces, plasticized samples only display a minor decrease of the stiffness (e.g., *E* of **TAB:Zn_5%** = 0.95 ± 0.13 GPa), while no statistically significant changes in the strain at break and toughness were observed (Figure 4a; Table 1). To further probe the influence of the **DIDP** addition on the mechanical properties, tensile tests were also performed above the *T*_g_ of the plasticized materials. To this end, stress–strain curves were recorded for the MSP containing 5 wt % plasticizer (**TAB:Zn_5%**) at 95 and 105 °C (Figures 4b and S12 and Table S3). Upon uniaxial tensile deformation at 95 °C, samples of **TAB:Zn_5%** display Young’s modulus of 0.44 ± 0.04 GPa, a tensile strength of 11 ± 1 MPa, and a strain at break of 5 ± 1%, while the neat **TAB:Zn** shows Young’s modulus of 0.78 ± 0.13 GPa, a tensile strength of 11 ± 1 MPa, and a strain at break of 2.0 ± 0.3% at this temperature. The toughness is thus considerably increased, from 131 ± 35 kJ m^–3^ for **TAB:Zn** to 366 ± 130 kJ m^–3^ for **TAB:Zn_5%**. As expected, increasing the temperature further to 105 °C for tensile testing does not lead to a significant change in the mechanical properties of the plasticized material.

To demonstrate the stimuli-responsive nature of the materials, the adhesive properties of neat **TAB:Zn** and of samples plasticized with 5 wt % of **DIDP** (**TAB:Zn_5%**) were explored in debonding-on-demand and re-bonding scenarios. Thus, thin films of the materials (thickness of ca. 100 μm) were placed on a stainless-steel substrate and heated to a temperature of 230 °C (see the Experimental Section for details). Upon melting, another stainless-steel substrate was placed on top of the MSP-covered area to create a lap joint with an overlap area of 10 × 10 mm^2^. After ca. 20 s, the lap joints were allowed to cool to room temperature and shear tests were carried out under ambient conditions. For samples freshly bonded with **TAB:Zn**, the measurements reveal a shear strength of 3.1 ± 0.3 MPa (Figure 4c) and the inspection of the dissociated joints reveals an adhesive failure mode (Figure S13; Table S4). The supramolecular nature of **TAB:Zn** allowed for re-bonding by locally heating (230 °C; 20 s) and re-joining the substrates. The re-bonded lap joints display a shear strength of 3.2 ± 0.4 MPa (Figures 4c and S14); i.e., the original bond strength was fully re-established. The measurements with **TAB:Zn_5%** reveal a shear strength of 2.6 ± 0.4 MPa for freshly prepared samples and of 2.8 ± 1.1 MPa for re-bonded lap joints (Figures 4d and S14); i.e., no statistically significant difference was observed in the room-temperature adhesive properties.

## Conclusions

In summary, a stiff and brittle semicrystalline MSP based on the trifunctional low-molecular-weight building block TAB and Zn(NTf_2_)_2_ was successfully plasticized by the addition of **DIDP**. The plasticizer is miscible with the supramolecular polymer in a concentration of up to ca. 5 wt %, and its incorporation reduces the *T*_g_ by more than 60 °C from 157 ± 1 to 90 ± 2 °C. At ambient temperature, i.e., below the *T*_g_, the plasticized MSP networks show a slightly lower stiffness but the same strength and toughness as the unplasticized material. When plasticized samples were deformed at temperatures above the *T*_g_, however, a significantly increased strain at break and toughness were observed. These findings show clearly that plasticization of a metallosupramolecular polymer is a promising strategy to straightforwardly tune the mechanical properties of these materials. For the investigated platform, it would be desirable to increase the miscibility of the two components, so that the *T*_g_ could be reduced further to below ambient temperature, which would potentially allow one to harness the toughening effect under more practically relevant conditions.

## Experimental Section

### Plasticizer Screening

To determine what plasticizers could be incorporated in the here presented MSP materials, different small molecules were investigated. Based on previously reported supramolecular polymers,^51^ dodecane was explored as a plasticizer but was not incorporated reliably into the MSP. Different phthalates were tested, including diethyl phthalate, dioctyl terephthalate, and **DIDP**, which were selected on account of their structural similarity to **TAB** and their relatively high boiling temperatures (299, 400, and 370 °C, respectively). While mixtures of **TAB:Zn** and diethyl phthalate and **DIDP** afforded transparent films, MSPs containing dioctyl terephthalate were turbid. Due to the higher boiling point of **DIDP** compared to diethyl phthalate, the former was chosen for in-depth experiments. Other phthalate derivatives were not investigated due to their reported toxicity.

### UV–vis Spectrophotometric Titrations

To demonstrate the complex formation, UV–vis absorption spectra were acquired during titrations of a **TAB** solution (*c* = 6.71 μM in CHCl_3_/CH_3_CN 9:1 *v*/*v*) with aliquots of 25 μL of a solution of Zn(NTf_2_)_2_ (*c* = 132 μM in CHCl_3_/CH_3_CN 9:1 *v*/*v*). The metal salt solutions were corrected for dilution effects by addition of an appropriate amount of the **TAB** solution. UV–vis spectra were also recorded during the titration of a solution of **TAB** containing 20 wt % of **DIDP** with respect to **TAB** with Zn(NTf_2_)_2_. Data were acquired upon addition of 25 μL aliquots of a solution of Zn(NTf_2_)_2_ (146 μM) and **TAB** (7.72 μM) in CHCl_3_/CH_3_CN (9:1 *v*/*v*) to a solution of **TAB** (7.72 μM) and **DIDP** in the same solvent mixture. Additionally, UV–vis spectra were recorded during the addition of a solution of **DIDP** (2418 μM) in CHCl_3_ to a solution of **TAB** (7.20 μM) and Zn(NTf_2_)_2_ with an M/L ratio of 1:2 in CHCl_3_/CH_3_CN (9:1 *v*/*v*), and spectra were recorded during the addition of a solution of Zn(NTf_2_)_2_ (143 μM) in CH_3_CN to a solution of **DIDP** (190 μM) in CHCl_3_.

### Metallosupramolecular Polymerization

A solution of Zn(NTf_2_)_2_ (113.82 mg, 0.18 mmol) in CH_3_CN was added to a solution of **TAB** (200 mg, 0.12 mmol) in CHCl_3_ and stirred until a clear solution was obtained. The solution containing the mixture of **TAB** and Zn(NTf_2_)_2_ was cast into a poly(tetrafluoroethylene) (PTFE) Petri dish, the solvent was evaporated overnight in a ventilated hood, and the sample was dried in vacuo at 50 °C over a course of 24 h to yield solvent-cast films of the MSP. For plasticized samples, a solution of the corresponding amount of plasticizer in CHCl_3_ was added to the solution containing the mixture of stoichiometric quantities of **TAB** and Zn(NTf_2_)_2_ and the mixture was stirred for 30 min. The solution containing all components was then cast into a PTFE Petri dish, the solvent was allowed to evaporate under ambient conditions in a ventilated hood, and plasticized samples were further dried in vacuo for 24 h at 50 °C.

### Processing

Solvent-cast samples of the MSP without or with a plasticizer were compression-molded in between Kapton sheets that were separated by a 250 μm thick aluminum spacer at a temperature of 220 °C for 45 s at a pressure of 6 t (0–5 wt % of plasticizer) or at 220 °C for 30 s at a pressure of 4 t (10–20 wt % of plasticizer) to obtain thin films with a thickness between 170 and 200 μm. The samples were typically cooled down to room temperature over a course of ca. 45 min by leaving them in between the steel plates of the hot press that were removed from the press for cooling. Quenched samples were processed at 230 °C for 45 s and a pressure of 6 t and immediately removed from the hot steel plates of the hot press and placed between cold ones for rapid cooling. Samples for adhesion tests were prepared in the same way, using a spacer with a thickness of 150 μm.

### Adhesive Properties

Adhesive properties were investigated in shear testing of single lap joints at room temperature. Two stainless-steel substrates were used for the preparation of single lap joints. One was heated on a hotplate to a temperature of 230 °C, and an area of ca. 10 × 10 mm^2^ was covered with a film of a thickness of around 100 μm of the MSP sample without or with a plasticizer. Upon melting of the film, the second lap joint was placed on top of the first one with an overlap area of the same size as the MSP film. The lap joints were left on the hotplate for ca. 20 s and then fixed with clamps and cooled down to room temperature. Samples prepared in this way were tested within 1 h after preparation. The same protocol was used to rebind the samples. Shear testing was carried out at a strain rate of 1 mm min^–1^ on a Zwick/Roell Z010 tensile tester that was equipped with a 10 kN load cell. All provided values are reported as the average of 3–4 independent experiments, and all errors given are standard deviations.

## Supporting Information Available

Schematic of the synthesis of TAB, UV-vis absorption spectra, TGA results, DSC results, and stress–strain curves (Figures S1–S14); overview of the thermal transitions, SAXS data, and mechanical properties (Tables S1–S4); detailed description of the synthetic procedures, materials, additional methods, and analytical data for all compounds; the source data of this study are available from the Zenodo repository at: https://doi.org/10.5281/zenodo.7306823 (PDF)
