# Supplementary material for: Plasticization of a Semicrystalline Metallosupramolecular Polymer Network
Source: ACS Polym Au. 2022 Nov 10;3(1):132–40. doi: 10.1021/acspolymersau.2c00044 (PMC9912337; doi:10.1021/acspolymersau.2c00044)
Supplement: Supplementary file 1 — lg2c00044_si_001.pdf [file lg2c00044_si_001.pdf]

Supporting Information

Plasticization of a Semicrystalline Metallosupramolecular Polymer Network

Franziska Marx,[1] Subhajit Pal,[1] Julien Sautaux,[1] Nazim Pallab,[1] Grégory Stoclet,[2]

Christoph Weder,[1,] and Stephen Schrettl[1,3*]*

[1] Adolphe Merkle Institute, University of Fribourg
Chemin des Verdiers 4, 1700 Fribourg, Switzerland

[2] Université de Lille, Unité Matériaux et Transformations
Cité scientifique, Bât. C6, 59655 Villeneuve d'Ascq, France

[3] Technical University of Munich, TUM School of Life Sciences
Maximus-von-Imhof-Forum 2, 85354 Freising, Germany

** To whom correspondence should be addressed:*

christoph.weder@unifr.ch

stephen.schrettl@unifr.ch

Table of Contents

1. Supporting Figures S1–S14 ... 2
2. Supporting Tables S1–S4 ... 16
3. Experimental Details ... 20
4. NMR Spectra ... 23
5. References ... 25

1. Supporting Figures S1–S14

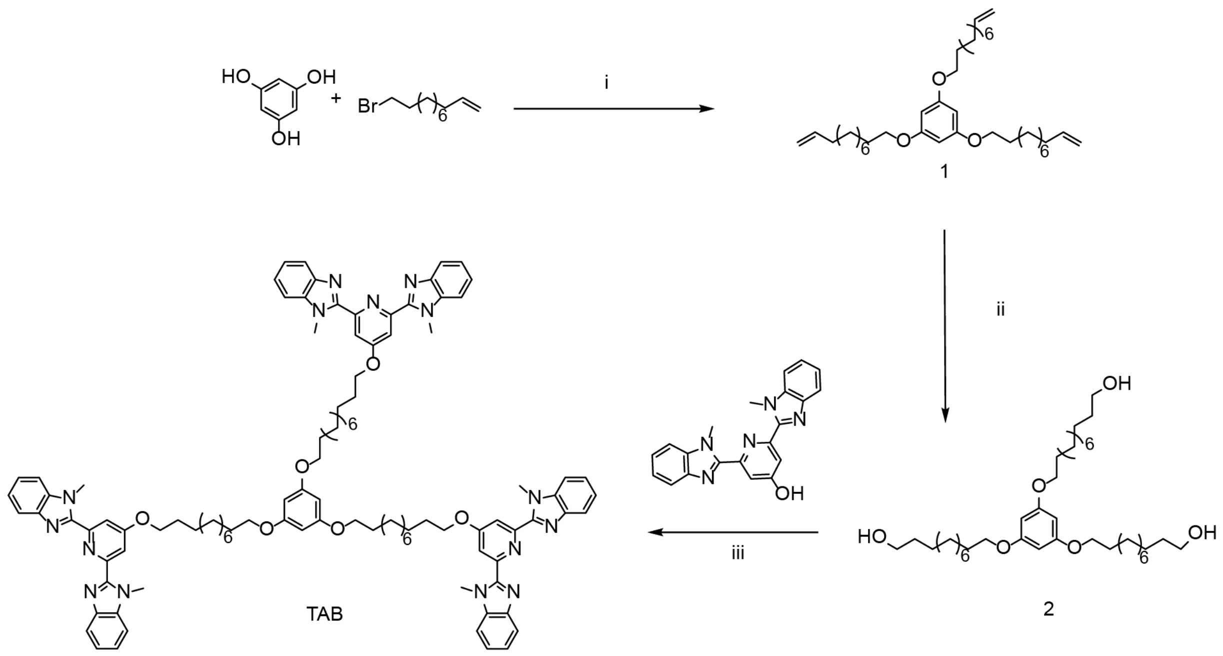

Supporting Figure S1. Schematic of the synthesis of **TAB**. The procedure was adapted from the previously reported synthetic route.[1] *Reagents and conditions: i)* K_2CO_3, KI, 18-crown-6, acetone, 24 h, reflux, 70 %; *ii)* 9-BBN, THF, 0 °C to r.t., 12 h; *then* H_2O_2, NaOH, water, 0 °C to r.t., 12 h, 85 %; *iii)* PPh_3, DEAD, THF, 0 to 50°C; 24 h, 60 %.

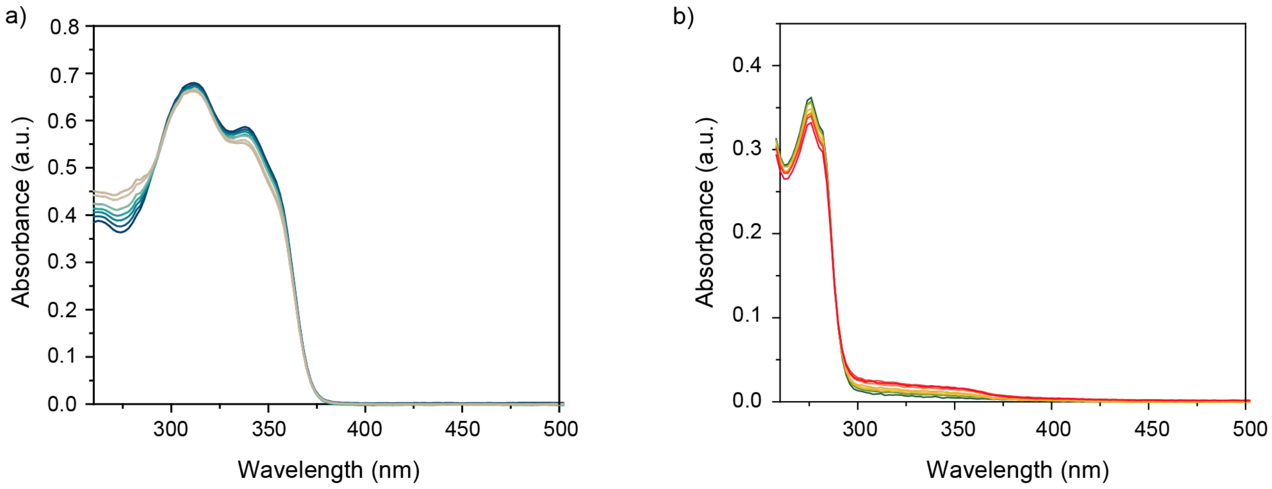

Supporting Figure S2. a) UV-vis absorption spectra recorded during the addition of aliquots (25 μL) of a solution of diisodecyl phthalate (**DIDP**; 2.42 mmol L^{-1}) in $CHCl_3$ to a solution containing a stoichiometric mixture of **TAB** and $Zn(NTf_2)_2$ with a metal-to-ligand ratio of 1:2 in a mixture of $CHCl_3/CH_3CN$ (7.20 μmol L^{-1}; 9:1 v/v). b) UV-vis absorption spectra recorded during the addition of aliquots (25 μL) of a solution of $Zn(NTf_2)_2$ (143 μmol L^{-1}) in CH_3CN to a solution of **DIDP** (190 μmol L^{-1}) in $CHCl_3$.

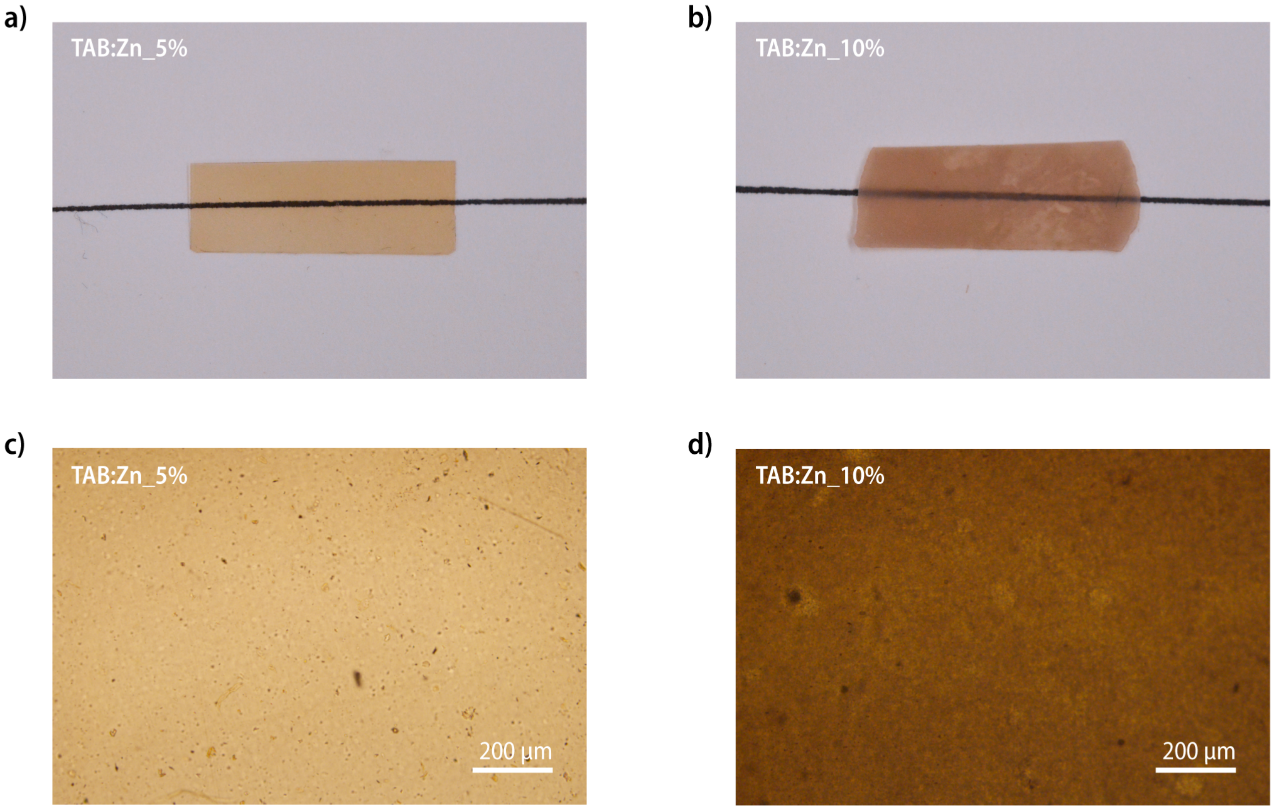

Supporting Figure S3. Comparison of (a,b) photographs and (c,d) optical microscopy images of samples of (a,c) **TAB:Zn_5%** and (b,d) **TAB:Zn_10%**. The plasticizer uptake into **TAB:Zn** appears to be limited to < 10 wt% of **DIDP**; and samples of **TAB:Zn_10%** that were prepared with a larger quantity of DIDP appear turbid and plasticizer sweating is observed.

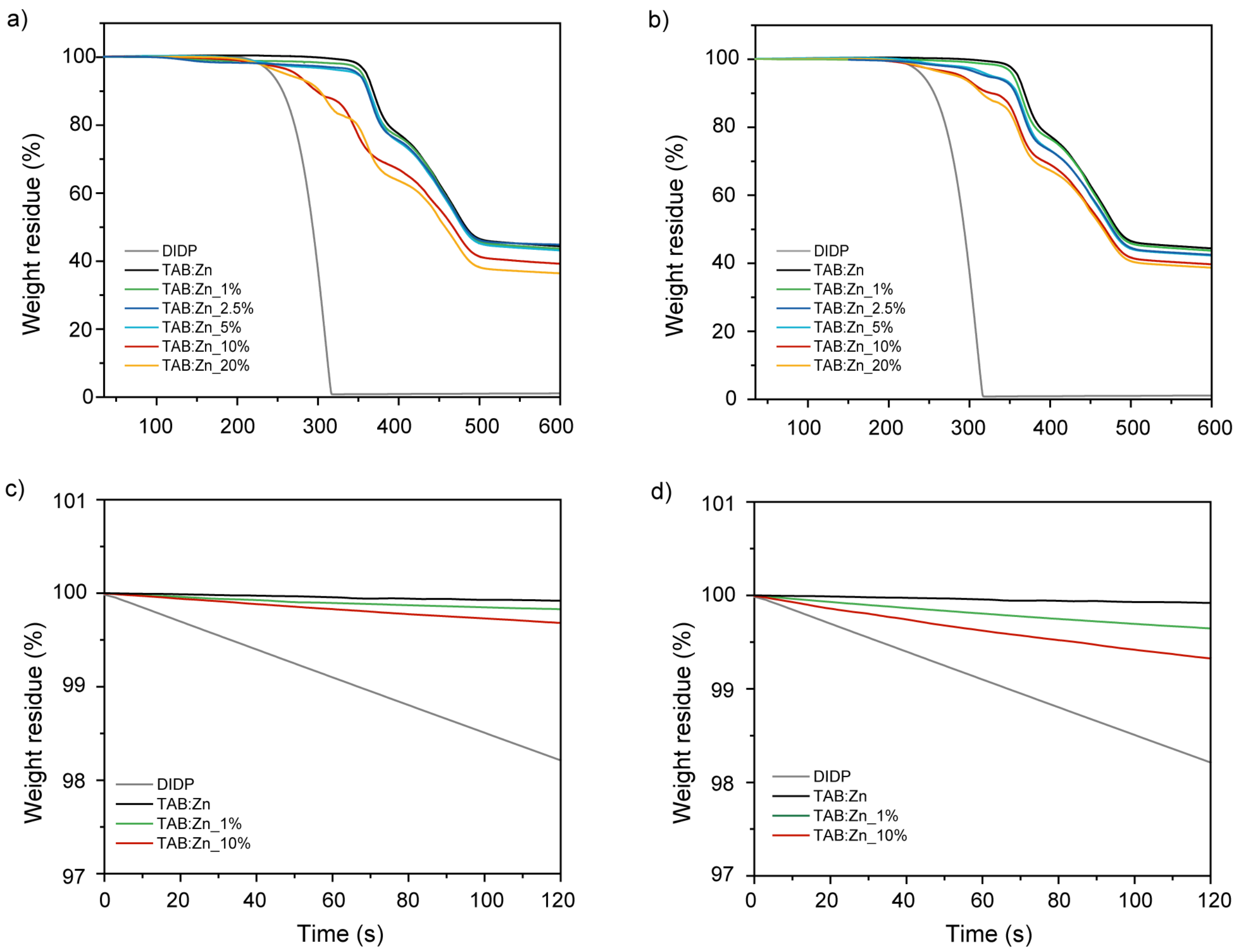

Supporting Figure S4. Thermogravimetric analysis (TGA) traces of diisodecyl phthalate (**DIDP**), the neat metallosupramolecular polymer **TAB:Zn**, and **TAB:Zn_1–20%**. a,b) TGA traces measured with a heating rate of 10 °C min^{-1} of (a) solvent-cast and (b) compression-molded samples. c,d) Plots of the weight loss recorded over time during isothermal measurements at a temperature of 220°C with (c) solvent-cast and (d) compression-molded samples.

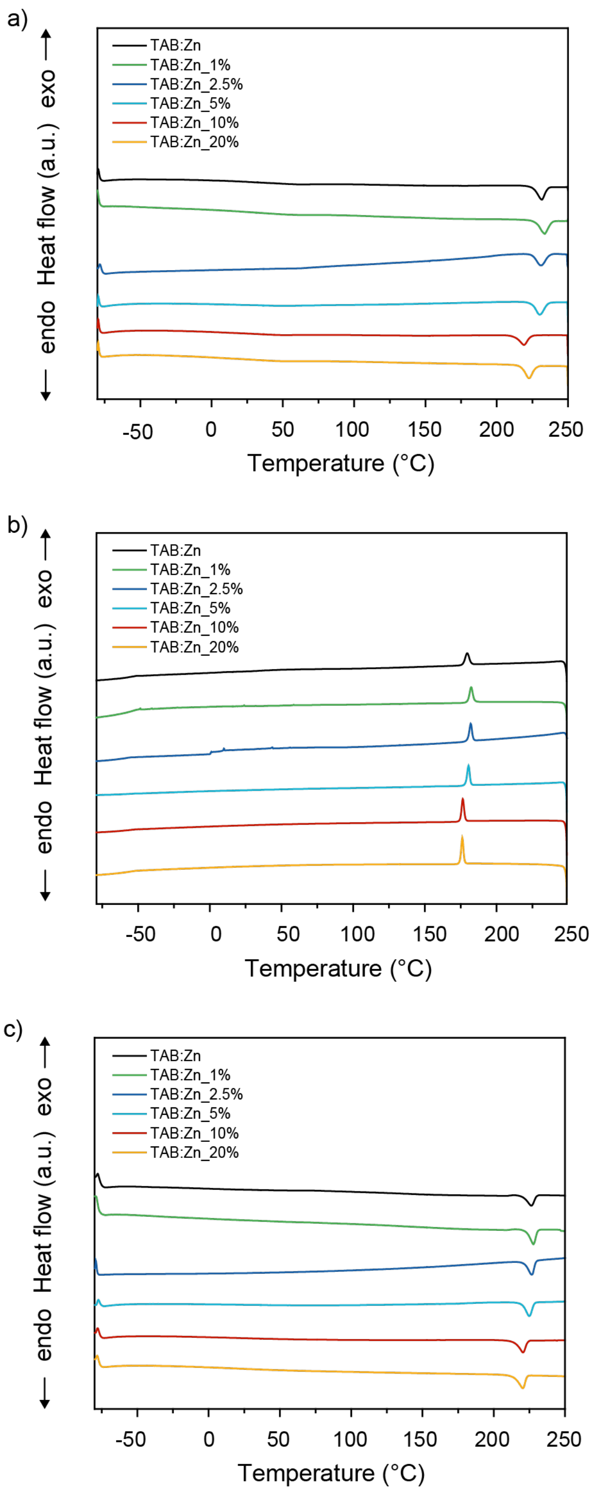

Supporting Figure S5. Differential scanning calorimetry (DSC) traces of compression-molded samples of **TAB:Zn** and **TAB:Zn_1–20%.** The plots show (a) the first heating, b) first cooling, and (c) the second heating traces measured with heating and cooling rates of 10 °C min^{-1}. Heating and cooling traces are vertically shifted for clarity.

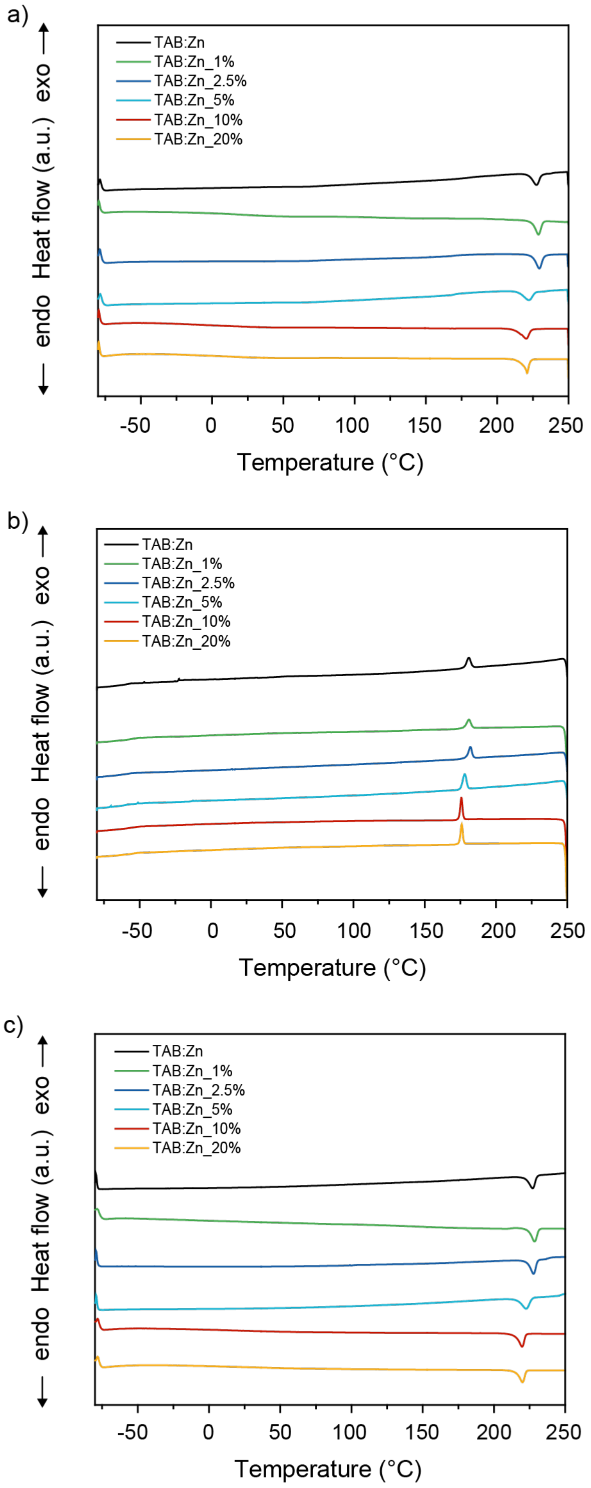

Supporting Figure S6. Differential scanning calorimetry (DSC) traces of solution-cast samples of **TAB:Zn** and **TAB:Zn_1–20%**. The plots show (a) the first heating, b) first cooling, and (c) the second heating traces measured with heating and cooling rates of 10 °C min^{-1}. Heating and cooling traces are vertically shifted for clarity.

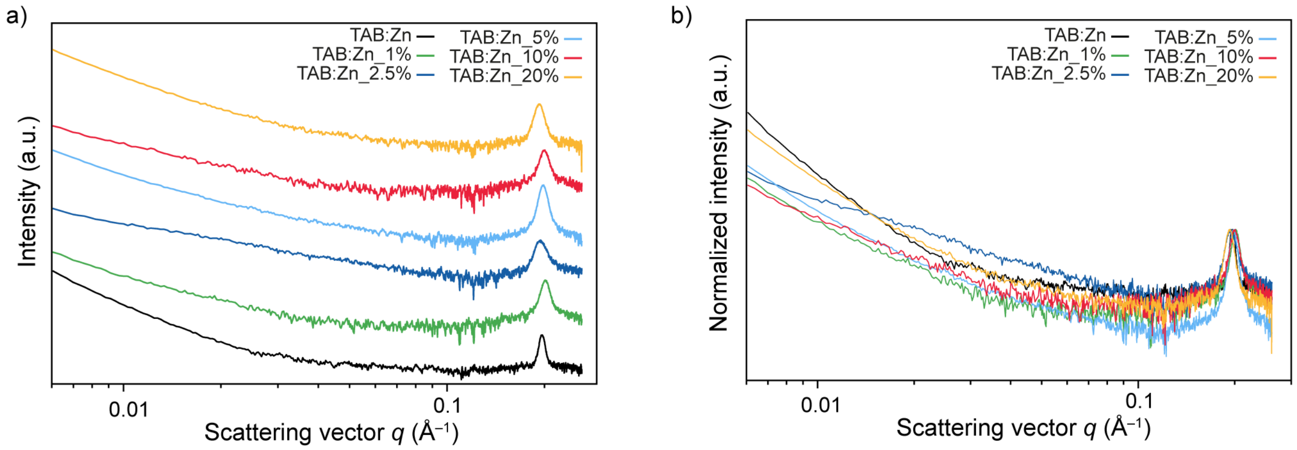

Supporting Figure S7. Comparison of the small-angle X-ray scattering (SAXS) profiles recorded in the range of 0.006 to ca. 0.25 Å^{-1} (sample-to-detector distance of ca. 2.5 m) of compression molded and slowly cooled samples of **TAB:Zn** and **TAB:Zn_1–20%**. Shown in (a) are the vertically shifted scattering profiles and in (b) the same profiles normalized to the intensity of the first order scattering peak at ca. 0.2 Å^{-1}. The SAXS measurements show no indication of microphase separation on larger length scales, even for samples of **TAB:Zn_10%** and **TAB:Zn_20%,** which showed plasticizer sweating.

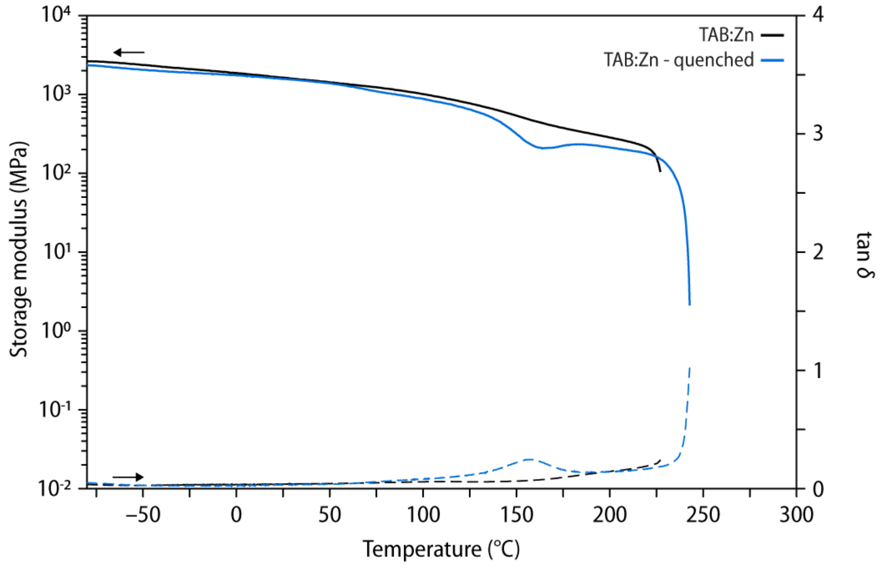

Supporting Figure S8. Dynamic mechanical analysis (DMA) traces of **TAB:Zn** samples that were slowly cooled (black trace) or quenched (blue trace) from the melt (solid lines: storage modulus, dashed lines: tan δ). For quenching experiments, compression-molded **TAB:Zn** films were placed in between Kapton sheets that were separated by 250 µm thick aluminum spacers. Following compression-molding at a temperature of 230 °C for 45 sec with a pressure of 6 t, samples were immediately removed from the hot steel plates and placed between cold (room temperature) steel plates for rapid cooling.

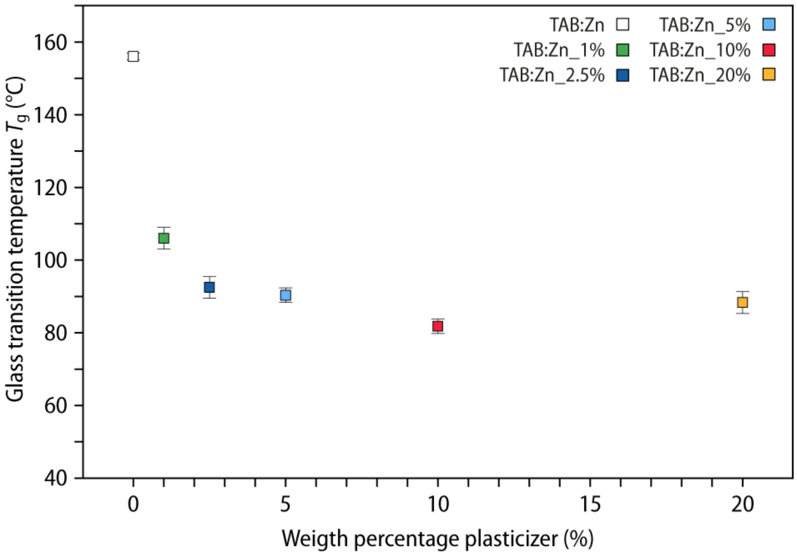

Supporting Figure S9. Plot of the glass transition temperature (T_g) against the plasticizer content for samples of **TAB:Zn** and **TAB:Zn_1–20%**. The T_g was determined from the local maxima in the tan δ traces of the DMA measurements. DMA measurements were carried out with a heating rate of 3 °C min^{-1}. The values represent averages of n = 2–6 individual measurements ± standard deviation. The T_g of samples of **TAB:Zn** was measured with samples that were quenched by rapid cooling from a temperature of 230 °C (see Experimental Details section).

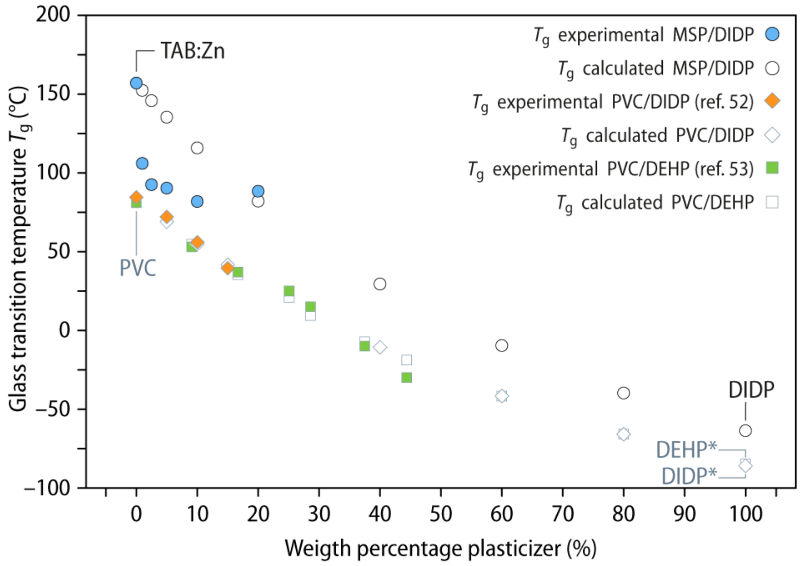

Supporting Figure S10. Plot of the experimentally determined and calculated glass transition temperature (T_g) against the weight fraction of added plasticizer for the metallosupramolecular polymer **TAB:Zn** and **TAB:Zn_1–20%** and reported literature values for poly(vinyl chloride) (PVC) plasticized with diisodecyl phthalate (DIDP)[2] or diisodecyl phthalate (DIDP).[2] The experimental values for **TAB:Zn** and **TAB:Zn_1–20%** were determined by DMA measurements and a reported value for DIDP that was determined by DMA measurements was used for the calculations with the Fox equation. The reported values for PVC with DIDP[2] were determined by DSC measurements[2] and for PVC with DEHP by dilatometry.[3] For all calculations for PVC/DIDP and PVC DEHP, previously reported T_g's for the plasticizers were used that were determined by DSC measurements (values marked with *).[4] For samples of PVC, the experimental values are in excellent agreement with the values calculated with the Fox equation.

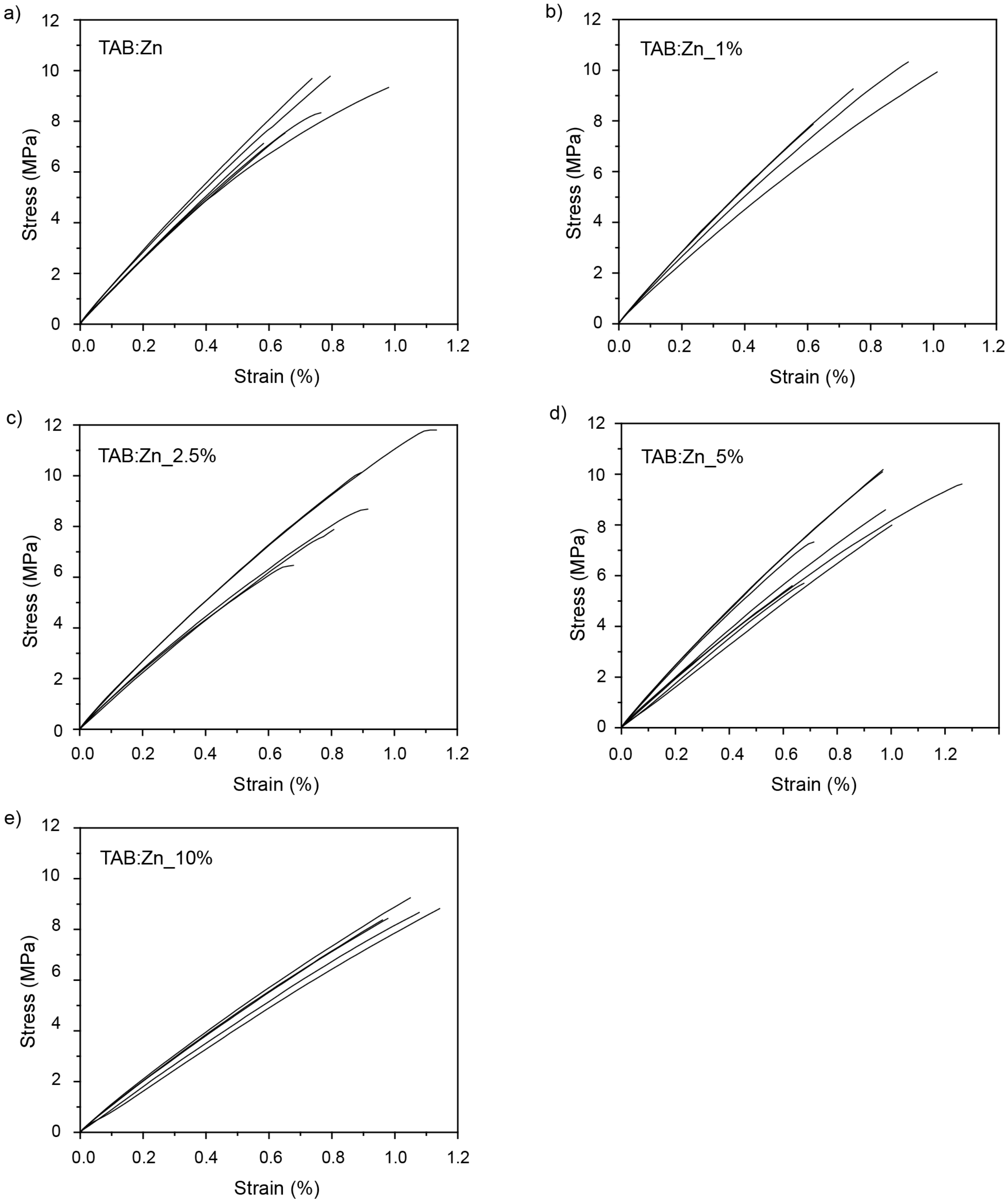

Supporting Figure S11. Stress-strain curves recorded during uniaxial tensile tests of **TAB:Zn** and **TAB:Zn_1-10%** at 25°C and at a strain rate of 1% min^{-1}.

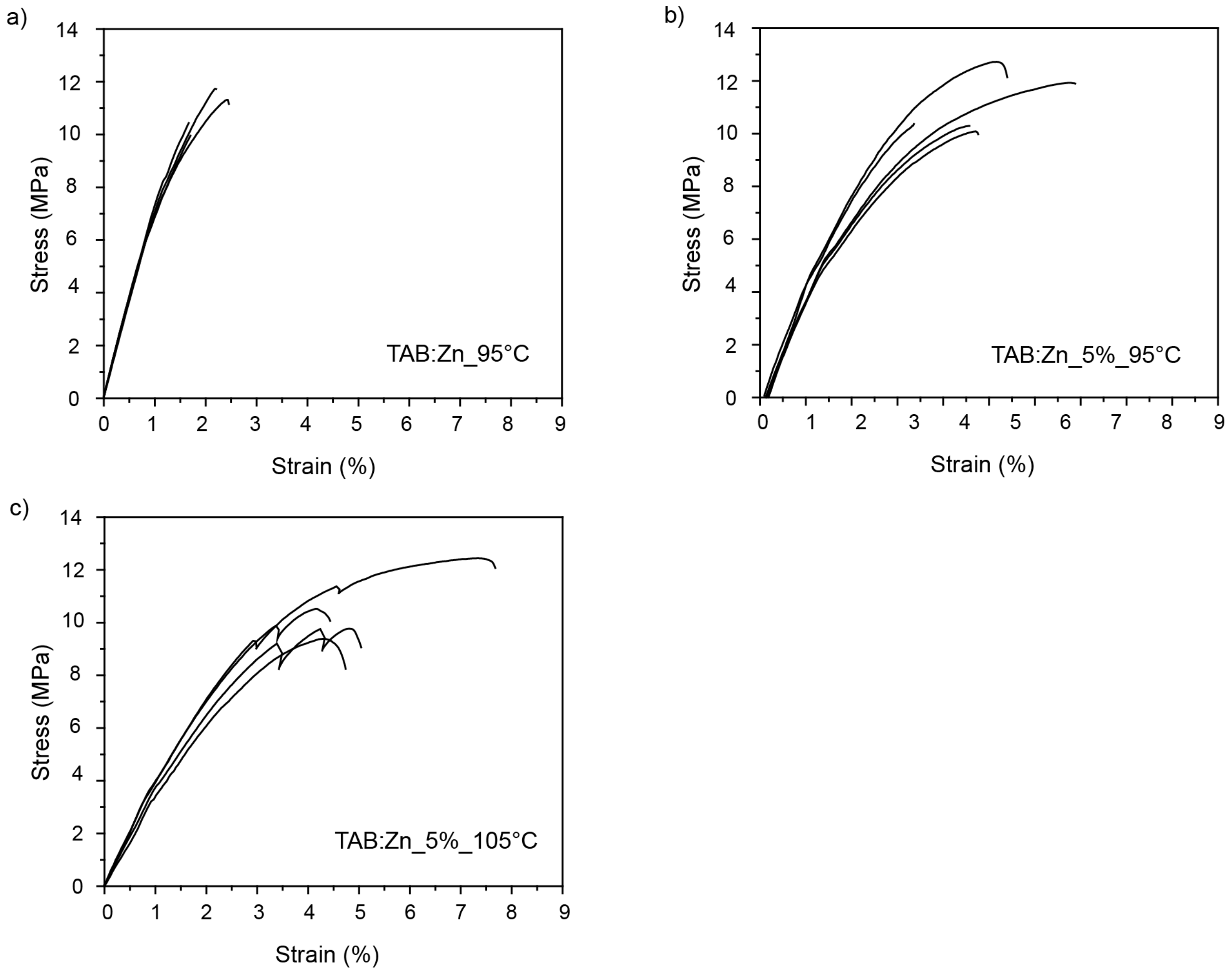

Supporting Figure S12. Stress-strain curves recorded during uniaxial tensile testing of **TAB:Zn** and **TAB:Zn_5%** at the indicated temperatures and at a strain rate of 1 % min^{-1}.

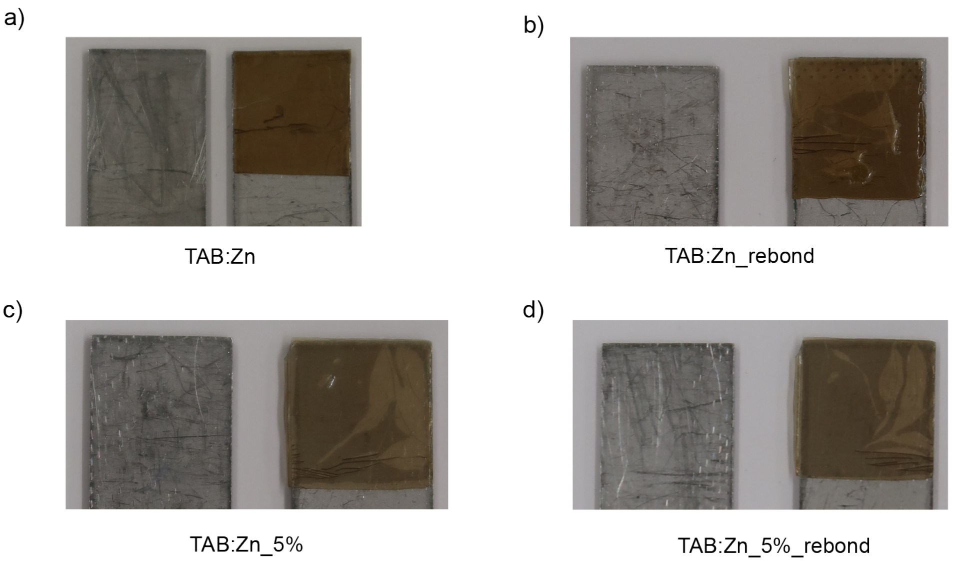

Supporting Figure S13. Photographs of stainless-steel single lap-joints after shear strength testing. a,b) Photographs of failed lap-joints of **TAB:Zn**-bonded samples after the (a) first and (b) second bonding and testing. c,d) Photographs of failed lap-joints of **TAB:Zn_5%**-bonded samples after the (c) first and (d) second bonding and testing.

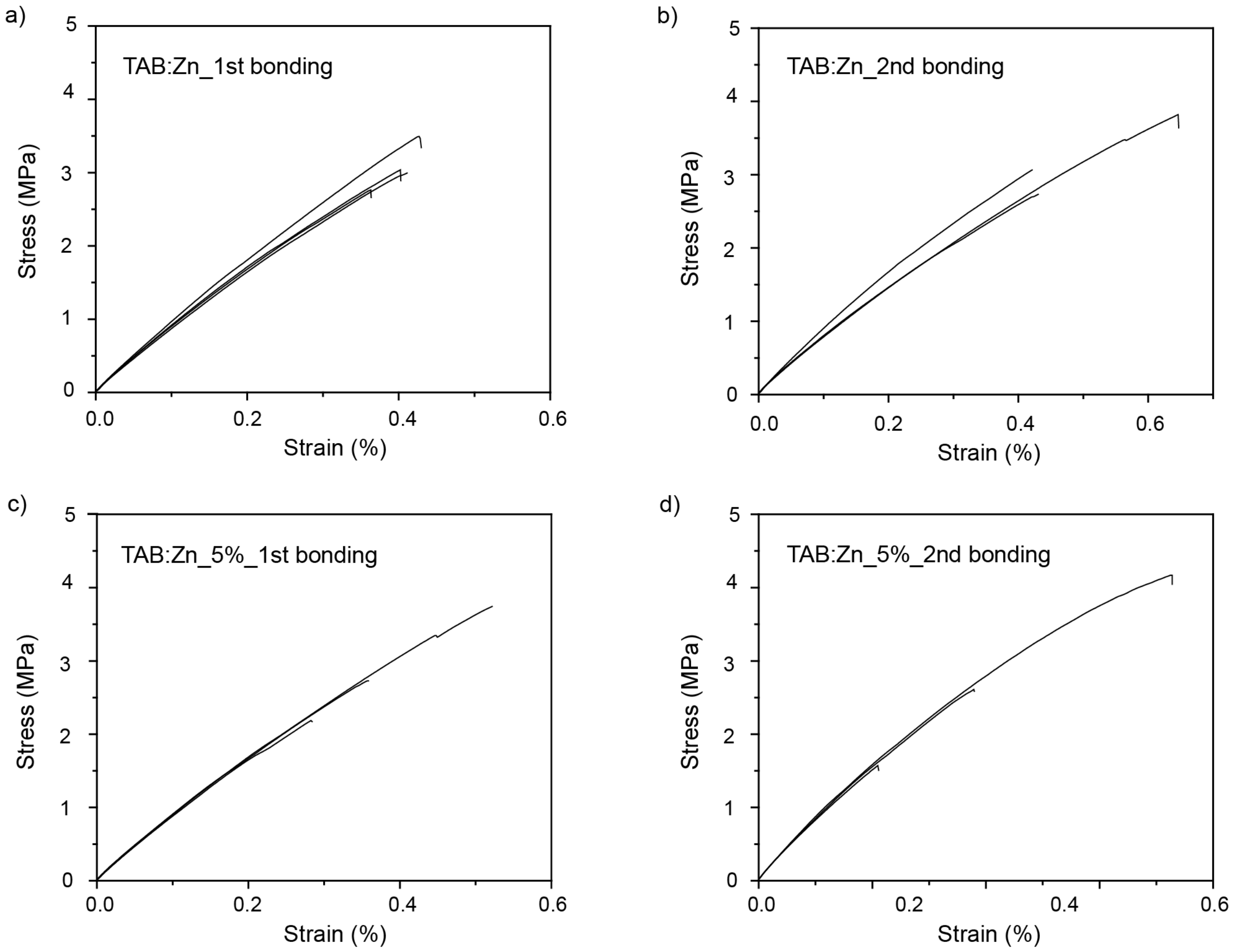

Supporting Figure S14. Shear tests of stainless-steel single lap-joints bonded with (a,b) **TAB:Zn** or (c,d) **TAB:Zn_5%.** Shown are the stress-strain curves recorded for freshly bonded (1st bonding) and re-bonded (2nd bonding) lap-joints.

2. Supporting Tables S1–S4

Supporting Table S1. Overview of the thermal transitions of the solution-cast (SC) and compression-molded (CM) samples of **TAB:Zn** and **TAB:Zn_1–20%** as determined by differential scanning calorimetry (DSC) with a heating rate of 10 °C min^{-1}.

Sample	First heating		First cooling		Second heating	
	T_m	ΔH_m	T_c	ΔH_c	T_m	ΔH_m
	(°C)	(W g^{-1})	(°C)	(W g^{-1})	(°C)	(W g^{-1})
CM – **TAB:Zn**	231	7.66	180	3.84	227	5.74
SC – **TAB:Zn**	228	5.91	180	3.37	226	6.24
CM – **TAB:Zn_1%**	233	8.26	182	4.30	228	6.69
SC – **TAB:Zn_1%**	229	6.19	181	3.28	228	5.49
CM – **TAB:Zn_2.5%**	232	7.57	182	3.98	227	6.79
SC – **TAB:Zn_2.5%**	230	7.25	182	3.42	228	6.43
CM – **TAB:Zn_5%**	230	8.17	181	4.34	225	6.88
SC – **TAB:Zn_5%**	222	6.22	178	4.40	223	6.81
CM – **TAB:Zn_10%**	219	6.32	176	4.58	221	6.60
SC – **TAB:Zn_10%**	220	6.31	176	4.08	220	5.95
CM – **TAB:Zn_20%**	223	7.53	176	4.72	220	6.97
SC – **TAB:Zn_20%**	221	6.00	176	3.58	220	5.31

Supporting Table S2. Overview of the SAXS data of **TAB:Zn** and **TAB:Zn_1-20%** samples at room temperature. All measured samples were processed by compression-molding and slow cooling.

**Sample**	***q*** ($Å^{-1}$)[a]	***L*** (nm)[b]	**FWHM** ($Å^{-1}$)[c]	**Grain size** (nm)[d]
**TAB:Zn**	0.197	3.19	0.020	28.0
**TAB:Zn_1%**	0.201	3.12	0.021	26.7
**TAB:Zn_2.5%**	0.198	3.17	0.021	27.6
**TAB:Zn_5%**	0.200	3.15	0.021	26.3
**TAB:Zn_10%**	0.196	3.21	0.022	25.2
**TAB:Zn_20%**	0.197	3.19	0.024	23.6

[a]q is the scattering angle of the first Bragg diffraction peak obtained from the center of a Pseudo Voigt function fit to this peak. [b]The period L is given as $2\pi/q$. [c]The full-width-at-half-maximum (FWHM) is given by the parameter of the Pseudo Voigt function fit. [d]The grain size is calculated using the Scherrer-analysis with 0.9 × (2π/FWHM).

Supporting Table S3. Mechanical properties of compression-molded **TAB:Zn** and **TAB:Zn_5%** films measured by uniaxial tensile testing at different temperatures. Data represent averages of n = 3–6 individual measurements ± standard deviation.

Sample	Young's Modulus E (GPa) [a]	Tensile strength σ_u (MPa) [a]	Strain at break ε_b (%) [a]	Toughness U_T (kJ m^{-3}) [a]
**TAB:Zn**	1.29 ± 0.07	9 ± 1	0.8 ± 0.1	36 ± 11
**TAB:Zn** at 95 °C	0.78 ± 0.13	11 ± 1	2.0 ± 0.3	131 ± 35
**TAB:Zn_5%**	0.95 ± 0.13	9 ± 1	0.9 ± 0.1	46 ± 15
**TAB:Zn_5%** at 95 °C	0.44 ± 0.04	11 ± 1	5 ± 1	366 ± 130
**TAB:Zn_5%** at 105 °C	0.40 ± 0.46	11 ± 2	5 ± 1	407 ± 169

[a]Measured by uniaxial stress-strain experiments at the indicated temperature with a strain rate of 1 % min^{-1}.

Supporting Table S4. Mechanical properties of single lap-joints bonded with **TAB:Zn** and **TAB:Zn_5%** as measured by shear testing. Data represent averages of *n* = 3 individual measurements ± standard deviation.

**Sample**	**Shear strain** (mm) [a]	**Shear stress** (MPa) [a]
**TAB:Zn**	0.40 ± 0.02	3.1 ± 0.3
**TAB:Zn** re-bonded	0.5 ± 0.1	3.2 ± 0.4
**TAB:Zn_5%**	0.39 ± 0.1	2.6 ± 0.4
**TAB:Zn_5%** re-bonded	0.44 ± 0.21	2.8 ± 1.1

[a]Data determined by stress-strain measurements in uniaxial tensile deformation mode with a strain rate of 1 mm min^{-1}.

3. Experimental Details

3.1 Instrumentation

NMR Spectroscopy. ^{1}H (400 MHz) and ^{13}C (100 MHz) NMR spectra were recorded on a Bruker A VIII HD spectrometer in $CDCl_3$. The spectra where calibrated on the residual solvent peak (7.26 ppm for $CDCl_3$ for ^{1}H NMR, 77.16 ppm for ^{13}C NMR). Data were evaluated with MestReNova (11.0), the chemical shifts δ are reported in part per million (ppm), coupling constants are reported in Hz.

UV-vis Spectroscopy. UV-vis absorption spectra were recorded on a Shimadzu UV-2401 PC spectrometer in $CHCl_3$/MeOH or $CHCl_3$/CH_3CN mixtures.

Polymer processing. Thin polymer films were prepared with a Carver CE Press.

Thermogravimetric analysis (TGA). Thermogravimetric analysis was performed in air using a Mettler-Toledo STAR instrument with a heating rate of 10 °C/min in a temperature range between 25 °C and 600 °C.

Differential scanning calorimetry (DSC). The measurements were performed on a Mettler-Toledo instrument STAR system under N_2 atmosphere with heating and cooling rates of 10 °C/min between –80 °C and 250 °C. The melting transition (T_m) is reported as the minimum of the major endothermic transition, the crystallization temperature (T_c) is reported as the maximum of the major exothermic transition.

SAXS/WAXS measurements. SAXS and WAXS measurements were performed on a Xeuss 2.0 apparatus (Xenocs) equipped with point collimator (beam size: 300×300 μm^2) and a micro source using Cu-K_α radiation (λ =1.54 Å). The sample-to-detector distance was calibrated using silver behenate as a standard and was around 35 cm or 2.5 m for SAXS and around 10 cm for WAXS measurements. Through view 2D diffraction patterns were recorded on a Pilatus 200k detector (Dectris). From the 2D patterns, integrated intensity profiles were computed using a Foxtrot® software.

Dynamic mechanical analysis (DMA). The measurements were conducted on a TA instruments Q800 DMA with a heating rate of 3 °C/min in a temperature range between –80 °C and 250 °C, a frequency of 1 Hz, and an amplitude of 3 μm using tensile clamps and rectangular shaped samples with dimensions of about 20 × 2.87 × 0.2 mm (l × w × t). All values are reported as the average of at least 3 independent measurements and all errors are reported as standard deviations. The same instrument and same sample geometries were used to perform tensile test on thin film samples with a pulling rate of 1% min^{-1} at room and elevated temperature.

3.2 Materials and Methods

All reactions were carried out in dried Schlenk glassware in an inert nitrogen atmosphere, if not stated otherwise. Spectroscopy grade $CHCl_3$ was purchased from Acros and before use purified from acid impurities by passage through a plug of activated (Brockman I) basic alumina. Zinc(II) trifluoromethanesulfonate

($Zn(NTf_2)_2$) (Strem Chemicals, Inc.) and all solvents (Sigma Aldrich or Acros) were used as received without further purification. 1,3,5-trihydroxybenzene, 11-Bromo-1-undecene, 9-borabicyclo[3.3.1]nonane (9-BBN), diethyl azodicarboxylate, triphenyl-phosphine, 18-Crown-6 ether, diethyl phthalate, dioctyl terephthalate, diisodecyl phthalate, dodecane and activated charcoal were purchased from Sigma Aldrich and used as received. Column chromatography was conducted on Geduran silica gel Si 60 from Merck (40-60 µm). Thin layer chromatography (TLC) was performed on TLC plates from Merck (Silica gel 60 F254). UV-light (254 nm) or potassium permanganate staining was used for detection.

3.3 Synthetic Procedures and Analytical Data

Synthesis of 1,3,5-tris(undec-10-en-1-yloxy)benzene (1). 1,3,5-Trihydroxybenzene (4 g, 31.7 mmol, 1 eq), K_2CO_3 (44 g, 317 mmol, 10 eq), catalytic amount of potassium iodide (100 mg), and 18-crown-6 (25g, 95.1 mmol, 3 eq) was added to a 500 mL two neck round bottom flask equipped with reflux condenser. The flask was evacuated and backfilled with N_2 three times. To the reaction mixture, dry acetone (250 mL) and 11-bromo-1-undecene (23.6 g, 101 mmol, 3.2 eq) were consecutively added. The resulting mixture was stirred and heated to reflux for 24 h. After cooling to room temperature, the solution was concentrated in vacuo, the crude residue was dissolved in water (200 mL), and the aqueous solution was extracted three time with EtOAc (20 mL). The combined organic layers were dried over $MgSO_4$, filtered, and concentrated in vacuo. Purification by column chromatography (DCM: hexane 1:4 to 2:3) furnished **1** as a colorless liquid (12.9 g, 22 mmol, 70 %).

^{1}H NMR ($CDCl_3$, 400 MHz) δ = 6.06 (s, 3H, 3 –C$H_{arom.}$), 5.82 (ddt, 3H, J = 17, 10, 7 Hz, 3 –CH=CH_2), 4.96 (m, 6H, 3 –CH=CH_2), 3.90 (t, 6H, J = 7 Hz, 3 –CH_2CH_2O-), 2.05 (m, 6H, 3 CH_2CH=CH_2), 1.75 (m, 6H, 3 –C$H_2$$CH_2$O-), 1.48–1.27 (m, 36H, 3 –CH_2(CH_2)$_6$$CH_2$–).

Synthesis of 11,11',11''-(benzene-1,3,5-triyltris(oxy))tris(undecan-1-ol) (2). 1,3,5-Tris(undec-10-en-1-yloxy)benzene (7.3 g, 12.5 mmol, 1 eq) was dissolve in dry THF (100 mL) and cooled to 0 °C. 9-Borabicyclo [3.3.1] nonane (9-BBN, 100 mL, 0.5 M in THF, 50.1 mmol, 4 eq) was added to this solution dropwise over the course of 40 min with a syringe pump. After complete addition, the mixture was allowed to warm to room temperature and stirred for 12 h. The mixture was cooled to 0 °C and water (7 mL) was added dropwise followed by NaOH (34 mL, 3 M) and aqueous hydrogen peroxide (34 mL, 30 %). The resulting mixture was allowed to warm to room temperature and stirred for 12h. The mixture was poured into a mixture of DCM (400 mL) and dilute aqueous HCl (5 %; 250 mL). The organic phase was separated, washed with brine, dried over $MgSO_4$, filtered, and concentrated in vacuo. Precipitated into cold diethyl ether furnished **2** (6.7 g, 10.6 mmol, 85 %) as a colorless solid.

^{1}H NMR ($CDCl_3$, 400 MHz) δ = 6.05 (s, 3H, 3 –CH_{arom}), 3.89 (t, 6H, J = 7 Hz, 3 –CH_2CH_2O–), 3.63 (t, 6H, J = 7 Hz, 3 –CH_2OH), 1.74 (quin, 6H, J = 7 Hz, 3 –C$H_2$$CH_2$O–), 1.56 (quin, 6H, J = 7 Hz, 3 –C$H_2$$CH_2$OH), 1.45–1.24 (m, 45H, 3 –CH_2(CH_3)$_7$$CH_2$– and 3 –$CH_2$OH).

Synthesis of 1,3,5-tris((11-((2,6-bis(1-methyl-1H-benzo[d]imidazol-2-yl)pyridin-4-yl)oxy)-undecyl)oxy)benzene (TAB). A mixture of **2** (1 g, 1.6 mmol, 1 eq), 4-hydroxy-2,6-bis(*N*-methylbenzimidazol-2'-yl)pyridine (2.8 g, 7.8 mmol, 5 eq), and triphenylphosphine (2 g, 7.8 mmol, 5 eq) was dissolved in dry THF (60 mL). The mixture was cooled to 0 °C and diethyl azodicarboxylate (DEAD; 1.36 g, 7.8 mmol, 5 eq, 40 wt% in toluene) was added dropwise. The reaction mixture was heated to 50 °C for 24 h under argon atmosphere. THF was removed under reduced pressure and the crude reaction mixture was diluted with DCM (10 mL). The solution was precipitated into cold diethyl ether (–20 °C, 500 mL) and the obtained pinkish solid was redissolved in DCM (100 mL) and stirred over activated charcoal (ca. 2 g, powder, mesh ~100) for 12 h. The mixture was filtered through a celite pad, and the solution was concentrated in vacuo. Precipitated into cold diethyl ether (-20 °C, 500 mL) furnished **TAB** (1.5 g, 9.4 mmol, 60 %) as a colorless solid.

^{1}H NMR (400 MHz, $CDCl_3$) δ = 7.96 (s, 6H, C-H_{arom}), 7.88 (m, 6H, C-H_{arom}), 7.46 (m, 6H, C-H_{arom}), 7.37 (m, 12H, C-H_{arom}), 6.05 (s, 3H, 3 –CH_{arom}), 4.23 (m, 24H, 6 –NCH_3 and 3 CH_2CH_2OMebip), 3.89 (t, 6H, J = 7 Hz, 3 –CH_2CH_2O–), 1.85 (quin, 6H, J = 7 Hz, 3 –C$H_2$$CH_2$OH), 1.74 (quin, 6H, J = 7 Hz, 3 –C$H_2$$CH_2$O–), 1.45–1.24 (m, 42H, 3 –CH_2(CH_3)$_7$$CH_2$– and 3 –C$H_2$OH). ^{13}C NMR (100 MHz, $CDCl_3$) δ = 166.7, 161.0, 151.0, 150.4, 142.4, 137.2, 123.6, 122.8, 120.1, 111.9, 109.9, 93.8, 77.4, 77.0, 76.7, 68.7, 68.0, 32.5, 29.6, 29.6, 29.5, 29.4, 29.3, 29.3, 28.9, 26.1, 25.9.

4. NMR Spectra

^1H NMR spectrum ($CDCl_3$, 400 MHz) of 1,3,5-tris(undec-10-en-1-yloxy)benzene (**1**).

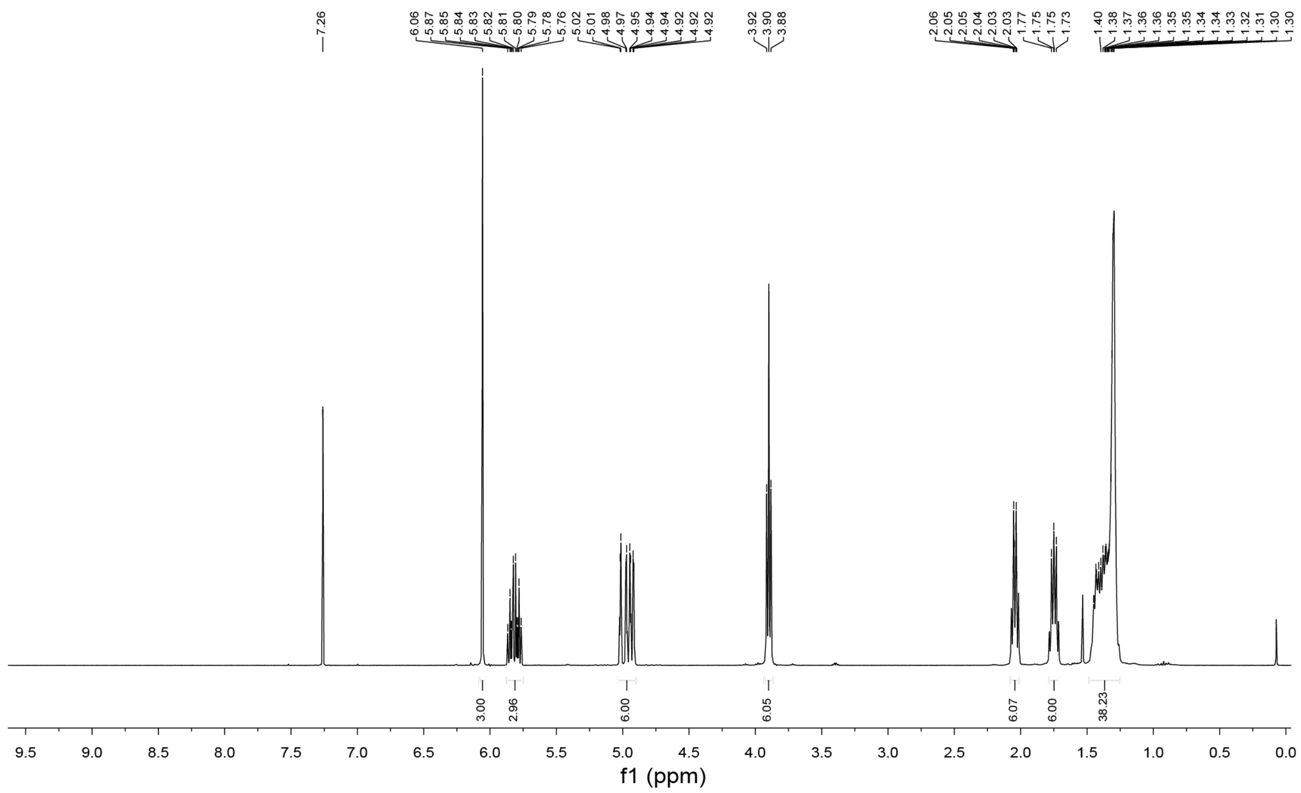

^1H NMR spectrum ($CDCl_3$, 400 MHz) of 11,11',11''-(benzene-1,3,5-triyltris(oxy))tris(undecan-1-ol) (**2**).

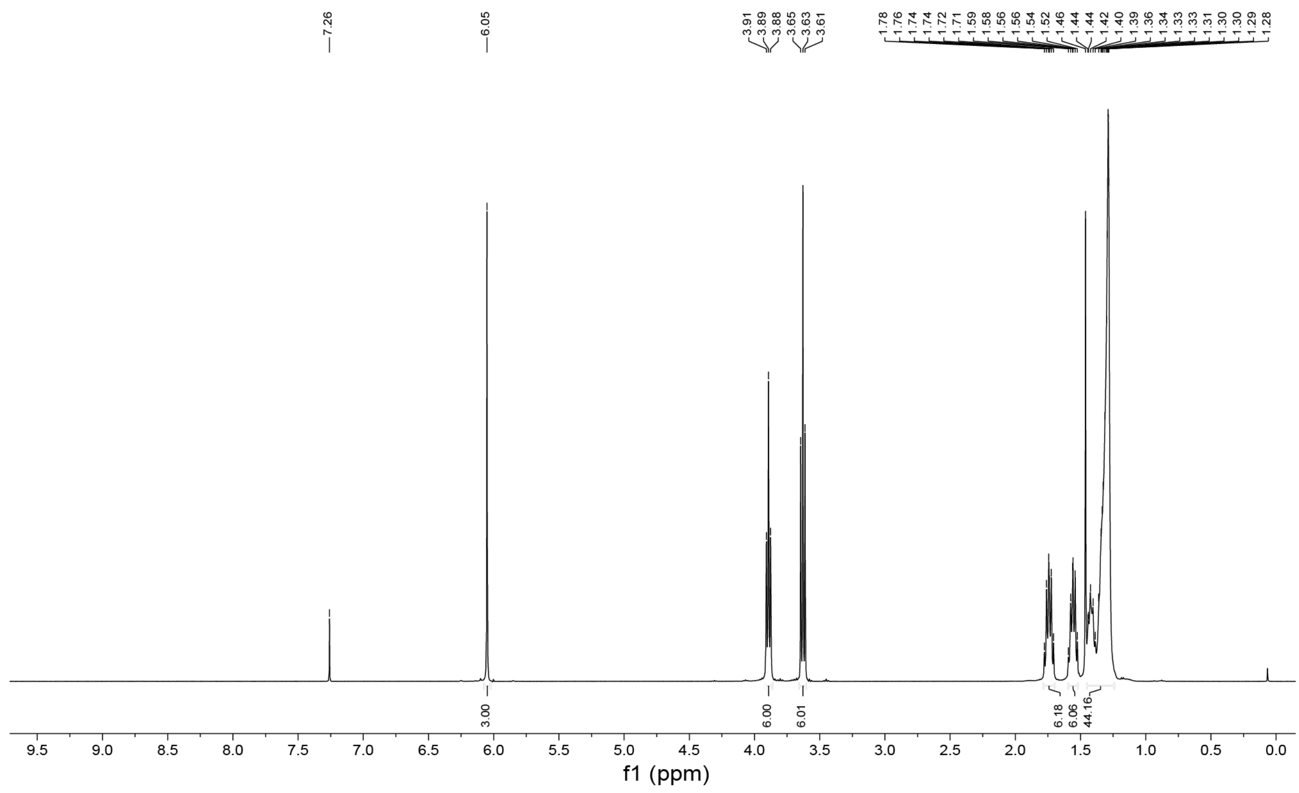

^{1}H NMR spectrum ($CDCl_3$, 400 MHz) of 1,3,5-tris((11-((2,6-bis(1-methyl-1H-benzo[d]imidazol-2-yl)pyridin-4-yl)oxy)-undecyl)oxy)benzene (**TAB**).

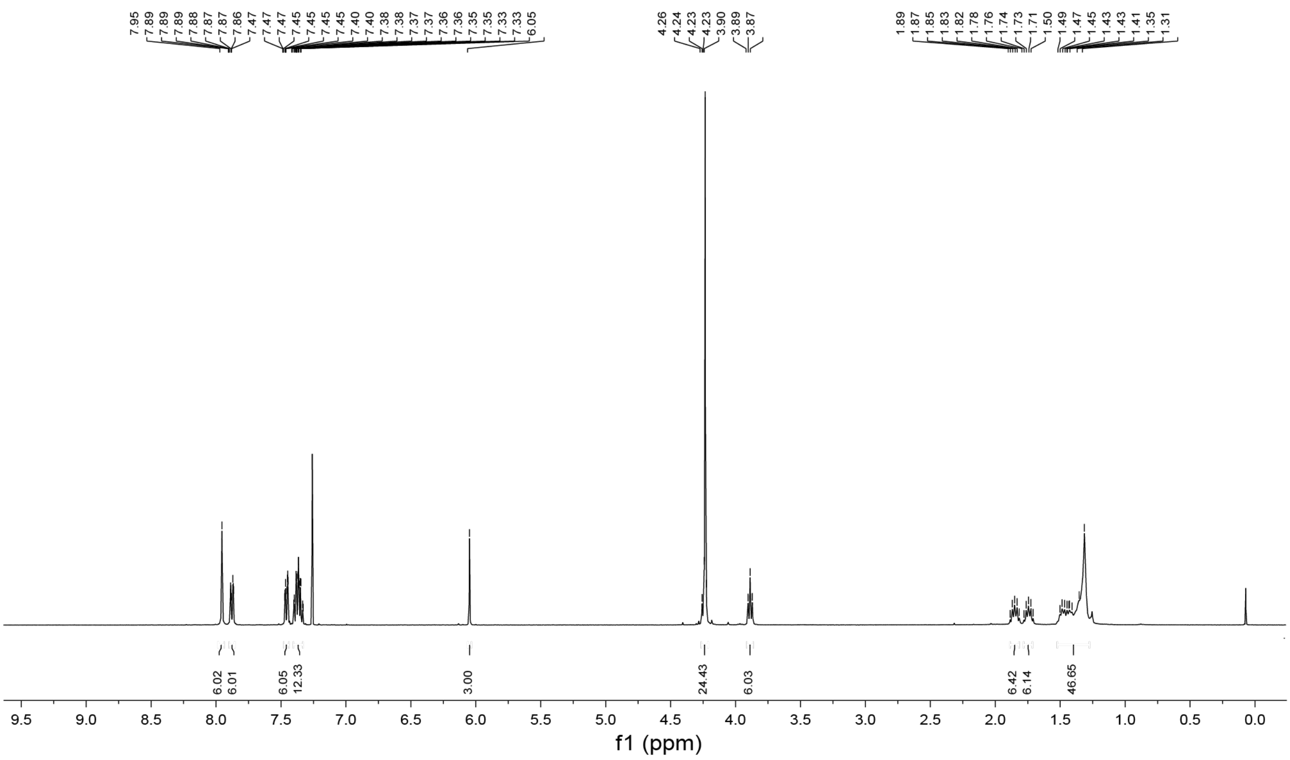

^{13}C NMR spectrum ($CDCl_3$, 100 MHz) of 1,3,5-tris((11-((2,6-bis(1-methyl-1H-benzo[d]imidazol-2-yl)pyridin-4-yl)oxy)-undecyl)oxy)benzene (**TAB**).

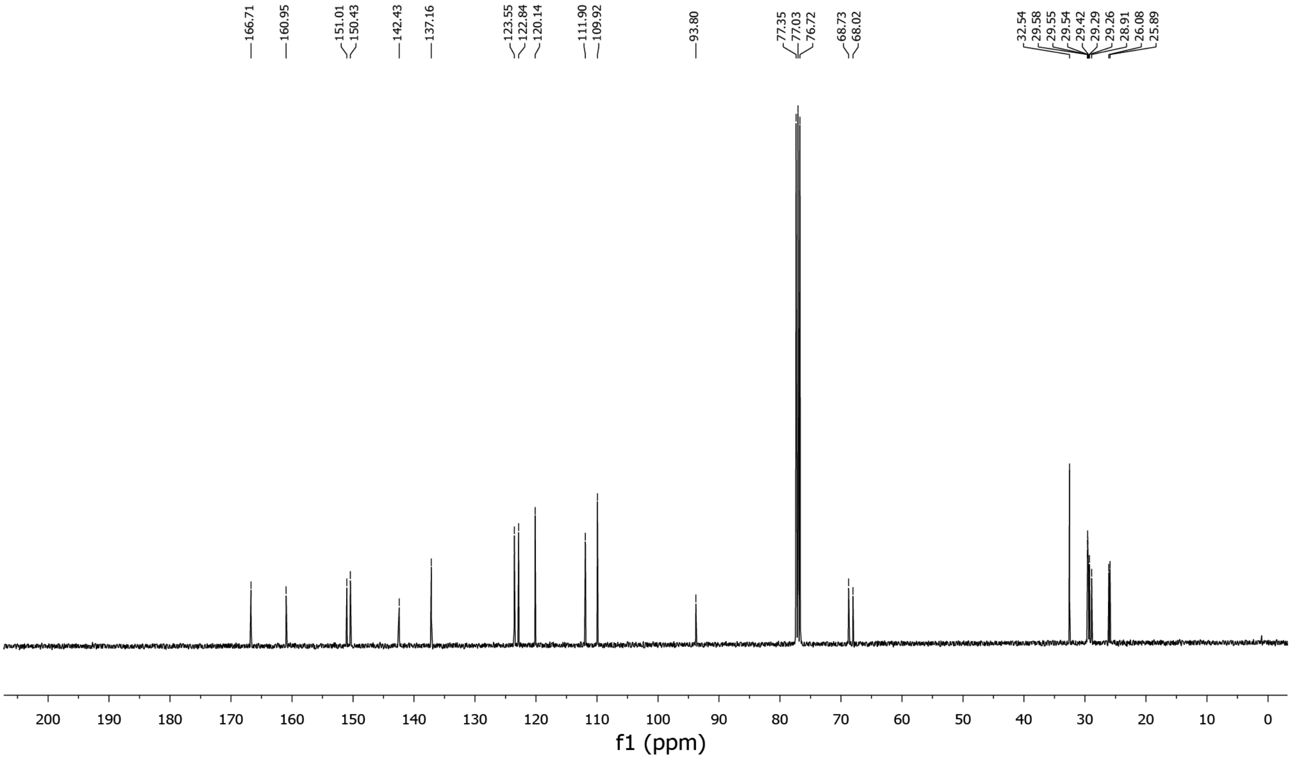

5. References

[1] J. Sautaux, F. Marx, I. Gunkel, C. Weder, S. Schrettl, *Nature Commun.* **2022**, *13*, 356.

[2] K. A. Mauritz, R. F. Storey, B. S. Wilson, *J. Vinyl Tech.* **1990**, *12*, 165–173.

[3] R. B. Taylor, A. V. Tobolsky, *J. Appl. Poly. Sci.* **1964**, *8*, 1563–1575.

[4] C. L. Ihemaguba, K. Marossy, *J. Therm. Anal. Calorim.* **2022**, *147*, 195–201.

[5] P. Cordier, F. Tournilhac, C. Soulié-Ziakovic, L. Leibler, *Nature* **2008**, *451*, 977–980.
